# Psychometric study of the brazilian version of the personality inventory for DSM-5–paper-and-pencil version

**DOI:** 10.3389/fpsyt.2022.976831

**Published:** 2022-09-13

**Authors:** Ana Maria Barchi-Ferreira, Flávia de Lima Osório

**Affiliations:** ^1^Department of Neurosciences and Behavior, Ribeirão Preto Medical School, University of São Paulo, São Paulo, Brazil; ^2^National Institute of Science and Technology for Translational Medicine, Brasília, Brazil

**Keywords:** personality, DSM-5, PID-5, psychometric properties, validity, reliability, scales

## Abstract

The Personality Inventory for DSM-5 - Self Reported Form (PID-5-SRF) operationalizes Criterion B of the personality alternative model of DSM-5 Section III and has already been cross-culturally adapted to many countries. The objective is to present evidence of validity and reliability of the Brazilian version of PID-5 (pencil-and-paper) in a Brazilian community sample. The sample was composed of 730 individuals from the general population [67.8% women, aged 33.84 (SD = ±15.2), 69.5% ≥ 12 years of schooling]. The participants were recruited in academic, organizational, healthcare, and business facilities in three Brazilian states. The snowball method was used. The PID-5 Brazilian version and the Revised NEO-Five Factor Inventory (NEO-FFI-R) were individually applied, and the retest was applied 30 days after. Satisfactory internal consistency (facets α ≥0.51; domains α ≥0.82) and test-retest reliability (facets ICC ≥ 0.45; domains ICC ≥0.76) were found, but a floor effect was verified in 97.7% of the items. Regarding convergent validity, strong correlations were found between the PID-5 and the NEO-FFI-R domains (r = −0.44 to 0.70). Ten facets did not fit the unidemensional structure. Confirmatory Factor Analyses did not present adequate goodness of fit, and Exploratory Analyses indicated that a five-factor model is more appropriate, though it presents some peculiarities concerning the original model. PID-5 also presented satisfactory goodness of fit to the personality hierarchical model. Generally, the instrument's psychometric indicators favor its use in the Brazilian context. However, some aspects demand attention, and more specific studies should be conducted to verify the impact of reverse-scored items, floor effect, and peculiarities of its internal structure (some facets' multidimensionality and interstitiality) concerning the original model.

## Introduction

The fifth edition of the Diagnostic and Statistical Manual of Mental Disorders [DSM-5; (1)] includes two diagnostic systems to classify personality disorders. One system is presented in section II, which maintains and updates the categories already consolidated in DSM-IV-TR (2). The other, presented in Section III, refers to a new dimensional model proposed by the DSM-5 Personality and Personality Disorders Workgroup.

The system presented in Section III comprises a hybrid dimensional and categorical model, which includes an assessment of the level of personality functioning (individual and interpersonal – Criterion A), pathological personality traits (Criterion B), pervasiveness and stability of functional impairment and pathological traits (Criteria C and D), and alternative explanations for personality pathology (Criteria E, F, and G). The guidelines for Criteria B were based on existing revised instruments and models assessing personality, besides discussions of the Workgroup previously mentioned, resulting in the Personality Inventory for DSM-5 [PID-5; (1)]. PID-5 comprises 220 items rated on a four-point Likert scale, distributed into 25 facets and five broader domains. The Negative Affect domain is characterized by intense and frequent unpleasant experiences such as anxiety, depression, guilt/shame, and worry, among others, along with behavioral manifestations. The Detachment domain concerns restricted emotional and social experiences, characterizing individuals who are low in extroversion. Antagonism is a domain represented by insensitivity, antisocial traits, grandiosity, and attention seeking. Finally, the Disinhibition domain is characterized by reckless, impulsive, and risky behavior, while Psychoticism is marked by strange and eccentric behavior and perception problems (1, 3).

There has been growing interest worldwide in using PID-5 since its first psychometric study (4). As a result, PID-5 has been subjected to cross-cultural adaptation in more than a dozen countries (United States, Canada, China, Italy, Germany, Portugal, Switzerland, Czech Republic, Denmark, Netherlands, Belgium, Spain, France, Norway, Australia, Singapore) and psychometric studies involving different (clinical and community) samples. Two important systematic literature reviews address a substantial portion (more than 80) of these studies (5, 6); generally, the results indicate satisfactory psychometric properties.

The reliability tests performed with Cronbach's alpha show satisfactory parameters for most facets and domains (>0.70). Although less frequently explored, temporal stability also showed appropriate indexes for periods between 1 week and 18 months (5, 6).

Convergent validity was tested against other instruments based on theoretical models of maladaptive personality traits, e.g., Computerized Adaptive Test of Personality Disorder (7), The Dimensional Assessment of Personality Pathology – Basic Questionnaire (8), but mainly the Five Factor Model (9), considering that PID-5 includes traits that correspond to a maladaptive variant of this model. In this context, various studies report strong correlations with the NEO Personality Inventory [NEO-FFI; (10)], a gold standard instrument to assess adaptive personality traits, and corroborate theoretically expected associations between Negative Affect and Neuroticism, Detachment and Extraversion, Antagonism and Agreeableness, and Disinhibition and Conscientiousness (5, 6, 11, 12).

Significant associations with psychopathology constructs (e.g., alcohol abuse, aggressiveness, and impulsivity, among others) and PID-5 ability to discriminate between clinical (considering different personality disorders) and non-clinical groups reinforce the instrument's clinical validity (13–16). However, its predictive ability (regarding the diagnostic criteria for Personality disorders according to Section II of DSM-IV/5) must be further explored (6).

Regarding its internal structure, studies have found it challenging to completely replicate the five-domain original model and the 25 facets proposed by Krueger et al. (4–6, 11, 12, 17, 18). The critical points include a lack of consensus on whether the facets are one-dimension (19, 20), interstitiality between facets [e.g., hostility - Labancz et al. (11)], and poor residual and comparative goodness of fit indexes (21), with facets not satisfactory loading on the domain of origin.

Furthermore, the five-level PID-5 hierarchical structure proposed by Wright et al. (22), ideally initiated with pathological personality advancing to the fifth level, composed of the five domains previously mentioned, also present some inconsistencies. These inconsistencies are observed from the third level, especially concerning the Disinhibition domain, which generally is not composed of the expected facets (17).

Even though these aspects have already been investigated and discussed, the PID-5 has the potential to be used in research and clinical settings, to favor the advancement of knowledge regarding personality disorders, and support the characterization of psychopathologies, screening potential cases, and assessing the progression of treatments (23–26). In this context, it is noteworthy that its psychometric properties have not been assessed for the Brazilian context; thus far, our research group has conducted its translation and cross-cultural adaptation (27). Therefore, this study aims to present validity (internal structure and convergent validity) and reliability (internal consistency and test/retest reliability) evidence of the Brazilian version of PID-5 (pencil-and-paper) in a Brazilian community sample. The hypothesis is that this instrument will present an internal structure that is appropriate to the original theoretical model (4) and psychometric indicators compatible with those presented in other cross-cultural studies (19, 28, 29).

## Methods

### Participants

A convenience sample was adopted, and the participants were recruited in academic, organizational, healthcare, and business facilities located in three Brazilian states in the northeast, midwest, and southwest. The research group individually approached the participants in the previously mentioned settings and invited them to participate in the study; snowball sampling or chain-referral sampling was adopted (30). No financial incentive was provided. Inclusion criteria were: being 18 years old or older, literate, and having good reading skills and text comprehension, regardless of gender. Not signing a free informed consent form or incorrectly completing or not completing any of the instruments were the exclusion criteria adopted. Data were collected between February and December 2019 using the pencil-and-paper version.

Of the 2,000 eligible individuals, 41.6% (*N* = 832) protocols were not returned, 2.9% (*N* = 58) were incorrectly completed, and 19.0% (*N* = 380) were excluded due to missing data. Hence, the final sample comprised 730 participants (36.5% were eligible). The Institutional Review Board approved the study (Process No. 4058/2018), and the participants signed free informed consent forms.

### Instruments

The data collection protocol consisted of the following instruments:

- Personality Inventory for DSM-5 (PID-5): developed by Krueger et al. (4) and adapted to the Brazilian Portuguese by Barchi-Ferreira et al. (27).
- Revised NEO-Five Factor Inventory (NEO-FFI-R – Short Version): developed by Costa and McCrae (31) and adapted and psychometrically assessed for the Brazilian context by Flores-Mendonza (32). It was designed to assess personality traits based on the Five-Factor Model. Its 60 items are distributed into five domains (i.e., Conscientiousness, Neuroticism, Extraversion, Agreeableness, and Openness to Experience) rated on a five-point Likert scale, ranging from “Strongly disagree” to “Strongly agree.”
- Socio-demographic and clinical form: the authors developed a 13-item, multi-choice, self-reported instrument.

### Data analysis

Data were coded according to technical guidelines and transferred to a database. The analyses were performed using IBM SPSS (33), R (34), and Mplus (35). The significance level was set at *p* ≤ 0.05 for all the analyses. Descriptive statistics were used to characterize the sample and analyze the items. Cronbach's alpha and McDonald's Omega (Ωt) were used to assess internal consistency, considering values above 0.70 (36). Intraclass Correlation Coefficient (ICC) was adopted to verify the test-retest reliability (from 15 days to 1 month) with a 95% confidence interval. The Spearman's correlation coefficient, interpreted according to the framework proposed by Streiner et al. (37), was used in the correlation analyses (item-facet, facet-domain, and convergent validity between the different domains/facets of both PID-5 and NEO-FFI-R).

The facets' unidimensionality was tested based on the polychoric correlation matrix and unweighted least squares (ULS) extraction (38, 39). Parallel analysis (40), Velicer's Map (41), and the Hull Method (42) were used to find the most appropriate number of factors for each facet. The adequacy of the one-factor solution was verified based on the Chi-Square test (X^2^), Tucker-Lewis Index (TLI), Root Mean Square Error of Approximation (RMSEA), and Root Mean Square Residual (RMSR), according to the following parameters: X^2^ [non-significant, X^2^/df bellow or equal to 3 (43, 44), TLI values close to 1.00 or higher than 0.90 and RMSR and RMSEA values close to or below 0.08 (43, 45).

The model proposed by Krueger et al. (4); 220 items distributed into five domains and 25 facets) was used as a reference in the Confirmatory Factor Analysis (CFA). The bootstrap resampling technique with a size replacement of 5000 was used for the sample to be sufficient to estimate the parameters. The Robust Maximum Likelihood (MLM) method was used for the extraction (46). The goodness of fit indexes previously mentioned were analyzed together with Standardized Root Mean Square Residual [SRMR – considered adequate when close to or below 0.08; (36)] and Comparative Fit Index [CFI – adequate when close to 1.00 or above 0.90; (45)].

Standardized regression weights (i.e., factor loadings) were calculated for the items in each of the facets and the facets in each of the instrument's domains; values equal to or above 0.30 were considered adequate (47). In addition, information concerning modification indexes was verified and used for the *post hoc* analyses to improve the model's goodness of fit.

Whenever the CFA results did not indicate the goodness of fit of the original model proposed by Krueger et al. (4) to the Brazilian data, Exploratory Factor Analysis (EFA) was performed. A series of factor analyses were performed at the facet level to explore the PID-5, considering Pearson's correlation matrix. The Unweighted Least Squares (ULS) method was used for the extraction with Promax rotation (38, 39). The same parameters used in the CFA were used to assess the models' adequacy.

Additionally, based on Goldberg (48), we calculated regression-based scores for each level (one to five) to explore the PID-5 hierarchical structure, which was then correlated to estimate the path coefficients between the hierarchy levels.

## Results

### Sample's sociodemographic characteristics

The final sample was composed of 730 participants; most were women (67.80%), aged 33.84 on average (SD = 15.15), with 12 or more years of schooling (69.50%). Approximately 31.00% lived with a partner, and 80.80% had a paid job. Only 13.80% of the sample presented health problems, predominantly hypertension and respiratory problems (34.3 and 26.2%, respectively), and 13.70% reported a psychiatric diagnosis: depression (61.7%) or anxiety (35.6%). Note that the sample excluded due to missing data does not significantly differ from the sample included regarding most sociodemographic and clinical variables, indicating no bias at this level. See Supplementary material SM1 for further information.

### Psychometric indicators

#### Analysis of items, facets, domains, and reliability

Considering the mean raw scores obtained by each of the items composing PID-5, the item with the highest score was “I rarely worry about things” (reverse-scored item – Mean = 2.37; SD = 0.88), and the item with the lowest score was “I sometimes hit people to remind them who is in charge” (Mean = 0.06; SD = 0.29). Note that the percentage of the answers “Very false or often false” was above 15% in almost all items (*N* = 215), characterizing a floor effect (49). Regarding the ceiling effect, only 23 items presented a percentage above 15% in the responses “Very true or often true.” These results are presented in detail in Supplementary material SM2, SM3.

The scores related to facets and domains are presented in Table 1, in which “Anxiousness” (Negative Affect) was the facet that obtained the highest score and “Callousness” (Antagonism) the lowest. Among the domains, Negative Affect scored the highest and Antagonism the lowest. Table 1 shows that 20 facets presented at least one item, the correlation of which with the total score was below the expected [ <0.50; (36)]. However, the same occurred for all the domains when their facets were observed. On the other hand, all the domains and most facets were correlated with the instrument's total score (above 0.50).

**Table 1:** Raw and weighted scores, distribution measures, correlations, and reliability indicators of PID-5 (paper-and-pencil) facets and domains, (*N* = 730).

**Do-main**	**Facets**	**No. of items**	**Distribution measures**					**Weighted score**		**Item-Facet correlation**	**Facet-total correlation**	**α**	**Ωt**	**T/R ICC (95%)**
			**Ass**	**SE**	**Kurt**	**SE**	**Norma-lity (#)**	**Mean**	**SD**					
**NA**	**Emotional lability**	7	0.38	0.09	−0.47	0.18	0.28	1.24	0.70	0.51–0.64	0.50	0.83	0.82	0.71 (0.55–0.82)
**NA**	**Anxiousness**	9	0.19	0.09	−0.73	0.18	0.63	1.48	0.67	0.17–0.71	0.62	0.84	0.85	0.75 (0.61–0.85)
**NA**	**Separation insecurity**	7	0.93	0.09	0.42	0.18	0.01	0.72	0.64	0.33–0.70	0.51	0.82	0.82	0.68 (0.51–0.80)
**NA**	**Submissiveness**	4	0.59	0.09	−0.19	0.18	0.09	0.86	0.67	0.52–0.59	0.45	0.75	0.77	0.63 (0.44–0.77)
**NA**	**Hostility**	10	0.56	0.09	−0.27	0.18	<0.001	0.88	0.60	0.38–0.67	0.65	0.84	0.85	0.71 (0.54–0.82)
**NA**	**Perseveration**	9	0.52	0.09	−0.25	0.18	0.18	0.84	0.56	0.38–0.61	0.74	0.80	0.81	0.69 (0.52–0.81)
**DET**	**Withdrawal**	10	0.98	0.09	0.51	0.18	0.02	0.73	0.62	0.52–0.74	0.61	0.88	0.89	0.79 (0.66–0.87)
**DET**	**Intimacy avoidance**	6	1.27	0.09	1.12	0.18	0.03	0.61	0.62	0.35–0.66	0.30	0.76	0.78	0.77 (0.63–0.86)
**DET**	**Anhedonia**	8	0.81	0.09	0.15	0.18	0.23	0.88	0.61	0.37–0.65	0.68	0.84	0.84	0.79 (0.66–0.87)
**DET**	**Depressivity**	14	1.63	0.09	2.49	0.18	<0.001	0.54	0.59	0.42–0.77	0.71	0.93	0.93	0.86 (0.76–0.91)
**DET**	**Restricted affectivity**	7	0.70	0.09	−0.02	0.18	<0.001	0.85	0.62	0.40–0.54	0.51	0.76	0.78	0.65 (0.47–0.78)
**DET**	**Suspiciousness**	7	0.23	0.09	−0.02	0.18	<0.001	1.18	0.47	−0.06–0.51	0.58	0.51	0.59	0.46 (0.22–0.64)
**ANT**	**Manipulativeness**	5	1.35	0.09	1.36	0.18	0.02	0.46	0.54	0.51–0.58	0.55	0.76	0.80	0.73 (0.58–0.83)
**ANT**	**Deceitfulness**	10	1.60	0.09	2.52	0.18	<0.001	0.41	0.44	0.19–0.65	0.59	0.81	0.85	0.82 (0.70–0.89)
**ANT**	**Grandiosity**	6	1.18	0.09	1.31	0.18	<0.001	0.62	0.54	0.34–0.55	0.46	0.70	0.73	0.57 (0.36–0.72)
**ANT**	**Attention seeking**	8	0.93	0.09	0.24	0.18	<0.001	0.64	0.60	0.46–0.73	0.54	0.86	0.87	0.65 (0.47–0.78)
**ANT**	**Callousness**	14	1.90	0.09	4.11	0.18	0.06	0.31	0.36	0.14–0.65	0.59	0.81	0.85	0.72 (0.55–0.83)
**DIS**	**Irresponsibility**	7	1.24	0.09	1.31	0.18	0.78	0.44	0.45	0.35–0.49	0.61	0.70	0.72	0.66 (0.47–0.78)
**DIS**	**Impulsivity**	6	0.76	0.09	0.10	0.18	0.06	0.83	0.67	0.47–0.70	0.49	0.85	0.86	0.61 (0.41–0.75)
**DIS**	**Distractibility**	9	0.55	0.09	−0.39	0.18	0.78	0.94	0.67	0.33–0.73	0.62	0.88	0.88	0.77 (0.63–0.86)
**DIS**	**Risk taking**	14	0.37	0.20	0.19	0.18	0.06	0.98	0.47	0.30–0.58	0.19	0.79	0.81	0.45 (0.21–0.64)
**DIS**	**Rigid perfectionism**	10	0.19	0.09	−0.69	0.18	0.01	1.12	0.66	0.46–0.70	0.36	0.86	0.86	0.71 (0.55–0.82)
**PSY**	**Unusual beliefs & experiences**	8	1.01	0.09	0.41	0.18	<0.001	0.60	0.55	0.34–0.57	0.56	0.76	0.76	0.69 (0.51–0.81)
**PSY**	**Eccentricity**	13	1.24	0.09	0.89	0.18	<0.001	0.62	0.67	0.64–0.78	0.72	0.94	0.95	0.86 (0.76–0.91)
**PSY**	**Perceptual dysregulation**	12	0.98	0.09	0.46	0.18	0.10	0.51	0.46	0.37–0.61	0.78	0.81	0.81	0.69 (0.53–0.81)
**Domains**														
**NA**		46	0.33	0.09	−0.35	0.18	0.11	1.00	0.48	0.07–0.67	0.74	0.82		0.86 (0.75–0.92)
**DET**		52	0.90	0.09	0.42	0.18	<0.001	0.76	0.45	−0.11–0.69	0.71	0.94		0.89 (0.82–0.94)
**ANT**		43	1.24	0.09	1.38	0.18	0.09	0.46	0.37	0.13–0.66	0.64	0.92		0.81 (0.69–0.89)
**DIS**		46	0.27	0.09	−0.44	0.18	<0.001	0.91	0.34	−0.01–0.54	0.74	0.87		0.76 (0.60–0.86)
**PSY**		33	0.98	0.09	0.34	0.18	<0.001	0.57	0.49	0.34–0.75	0.77	0.93		0.86 (0.76–0.92)

ANT, Antagonism; Asy, Asymmetry; DET, Detachment; DIS, Disinhibition; SD, Standard Deviation; SE, Standard Error; ICC, Intraclass Correlation Coefficient; Kurt, Kurtosis; NA, Negative Affect; PSY, Psychoticism; T/R, Test-Retest; α, Cronbach's alpha; Ωt, McDonald's Omega; #, Assessment of normality: Shapiro-Wilk test.

The total scale's internal consistency was α = 0.98/ (Ωt = 0.98). Individually, all the facets presented adequate values (α and Ωt > 0.70), except Suspiciousness (α = 0.51/ Ωt = 0.59), while alpha values for the domains were above 0.87. Test-retest reliability was verified for each item individually, and the indicators were above 0.50 for 96 items (see Supplementary material SM2). Regarding facets and domains, the indexes were considered strong/very strong (> 0.50) except for the Suspiciousness (0.46) and Risk-Taking (0.45) facets.

#### Convergent validity indicators

NEO-FFI was used to estimate the PID-5 convergent validity. The results confirm significant correlations, predominantly of moderate/strong magnitude, between the expected domains: Negative Affect and Neuroticism (*r* = 0.70), Detachment and Extroversion (*r* = −0.59), Antagonism and Agreeableness (*r* = −0.64), Disinhibition and Conscientiousness (*r* = −0.44), Psychoticism and Neuroticism (*r* = 0.43), and Psychoticism and Openness to Experience (*r* = 0.14). Apart from that, the facets theoretically related to PID-5 different domains were also those more strongly and significantly correlated with NEO-FFI domains (facets that compose Negative Affect *vs*. Neuroticism: *r* ≥ 0.41; Detachment vs. Extroversion *r* ≥ −0.19; Antagonism *vs*. Agreeableness *r* ≥ −0.35, and Disinhibition vs. Conscientiousness *r* ≥ −0.20). Further details are presented in Table 2 and Supplementary material SM4.

**Table 2:** PID-5 (paper-and-pencil) convergent validity indicators using NEO-FFI as reference (*N* = 730).

**PID-5 domains**							**NEO-FFI domains**				
**Domains**	**Facets**	**NA**	**DET**	**ANT**	**DIS**	**PSY**	**NEU**	**EXTR**	**OPEN**	**AGR**	**CONS**
**NA**	**Emotional lability**	0.75*	0.33*	0.21*	0.45*	0.46*	0.51*	−0.05	0.13*	−0.09*	−0.22*
**NA**	**Anxiousness**	0.84*	0.54*	0.30*	0.47*	0.48*	0.70*	−0.26*	0.04	−0.19*	−0.24*
**NA**	**Separation insecurity**	0.72*	0.36*	0.35*	0.38*	0.38*	0.44*	−0.06	−0.09*	−0.19*	−0.23*
**NA**	**Submissiveness**	0.53*	0.38*	0.34*	0.36*	0.31*	0.41*	−0.09*	0.08*	−0.11*	−0.30*
**NA**	**Hostility**	0.71*	0.53*	0.58*	0.55*	0.47*	0.46*	−0.23*	−0.13*	−0.60*	−0.29*
**NA**	**Perseveration**	0.79*	0.61*	0.49*	0.62*	0.63*	0.50*	−0.22*	−00.02	−0.33*	−0.34*
**DET**	**Withdrawal**	0.47*	0.84*	0.39*	0.42*	0.51*	0.38*	−0.67*	−0.16*	−0.38*	−0.31*
**DET**	**Intimacy avoidance**	0.13*	0.55*	0.20*	0.18*	0.28*	0.12*	−0.30*	–.14*	−0.17*	−0.13*
**DET**	**Anhedonia**	0.58*	0.86*	0.33*	0.42*	0.51*	0.58*	−0.59*	–.19*	−0.30*	−0.48*
**DET**	**Depressivity**	0.66*	0.85*	0.41*	0.51*	0.63*	0.65*	−0.42*	0.02	−0.29*	−0.51*
**DET**	**Restricted affectivity**	0.30*	0.67*	0.43*	0.41*	0.44*	0.15*	−0.34*	−0.22*	−0.36*	−0.25*
**DET**	**Suspiciousness**	0.55*	0.58*	0.42*	0.44*	0.46*	0.42*	−0.19*	−0.08*	−0.36*	−0.24*
**ANT**	**Manipulativeness**	0.38*	0.32*	0.84*	0.48*	0.46*	0.20*	−0.02	0.10*	−0.52*	−0.33*
**ANT**	**Deceitfulness**	0.40*	0.43*	0.85*	0.51*	0.48*	0.27*	−0.13*	0.03	−0.57*	−0.48*
**ANT**	**Grandiosity**	0.35*	0.27*	0.71*	0.38*	0.43*	0.07	−0.03	−0.00	−0.41*	−0.09*
**ANT**	**Attention Seeking**	0.50*	0.28*	0.75*	0.50*	0.44*	0.27*	0.11*	0.15*	−0.35*	−0.27*
**ANT**	**Callousness**	0.37*	0.55*	0.77*	0.48*	0.50*	0.20*	−0.28*	−0.20*	−0.63*	−0.37*
**DIS**	**Irresponsibility**	0.43*	0.48*	0.59*	0.65*	0.52*	0.30*	−0.17*	−0.04	−0.39*	−0.61*
**DIS**	**Impulsivity**	0.52*	0.31*	0.36*	0.65*	0.38*	0.38*	−0.02	−0.06	−0.30*	−0.38*
**DIS**	**Distractibility**	0.61*	0.53*	0.42*	0.70*	0.51*	0.49*	−0.22*	−0.06	−0.26*	−0.58*
**DIS**	**Risk taking**	0.02	0.09*	0.35*	0.55*	0.28*	−0.04	0.09*	0.01**	−0.24**	−0.20**
**DIS**	**Rigid perfectionism**	0.45**	0.30**	0.21**	0.46**	0.35**	0.19**	−0.13**	0.03	−0.06	0.22**
**PSY**	**Unusual beliefs & experiences**	0.44**	0.39**	0.44**	0.50**	0.79**	0.22**	−0.12**	0.11**	−0.22**	−0.16**
**PSY**	**Eccentricity**	0.53**	0.65**	0.55**	0.58**	0.92**	0.38**	−0.30**	0.16**	−0.38**	−0.39**
**PSY**	**Perceptual dysregulation**	0.69**	0.61**	0.54**	0.65**	0.87**	0.47**	−0.21**	0.05	−0.32**	−0.39**
	**DOMAINS**										
							**NEU**	**EXTR**	**OPEN**	**AGR**	**CONS**
	**NA**	**–**					0.70*	−0.22*	−0.01	−0.38*	−0.36*
	**DET**	0.64*	**–**				0.56*	−0.59*	−0.15*	−0.41*	−0.47*
	**ANT**	0.52*	0.49*	**–**			0.27*	−0.10*	0.01	−0.64*	−0.41*
	**DIS**	0.65*	0.55*	0.61*	**–**		0.41*	−0.14*	0.01	−0.39*	−0.44*
	**PSY**	0.63*	0.66*	0.60*	0.66*	–	0.42*	−0.26*	0.14*	−0.37*	−0.39*

AGR, Agreeableness; ANT, Antagonism; CONS, Conscientiousness; DIS, Disinhibition; DET, Detachment; EXTR, Extroversion; ICC, Intraclass Coefficient Correlation; NA, Negative Affect; NEU, Neuroticism; OPEN, Openness to Experience; PSY, Psychoticism; α, Cronbach's alpha; ^*^p ≤ 0.05; Bold, ≥ 0.51 (Strong correlations according to the parameters established by 30).

#### Validity indicators based on the internal structure

##### Facets' unidimensionality

The different methods used to estimate the number of factors associated with the facets indicate that many are not unidimensional. This fact was corroborated by the goodness of fit analysis associated with the one-factor model, which was also unsatisfactory. Table 3 and Supplementary material SM5 indicate that a two-dimension structure is more suitable for Emotional Lability, Anxiousness, Hostility, Perseveration, Depressivity, Suspiciousness, Attention Seeking, Risk-Taking, Unusual Beliefs & Experiences, and Perceptual Dysregulation. The goodness of fit of the Restricted Affectivity, Deceitfulness, and Callousness facets did not improve when one-dimension models were tested.

**Table 3:** Unidimensionality analysis of PID-5 (paper-and-pencil) facets according to different methods (*N* = 730).

**Domains**	**Facets**	**Number of factors suggested by the method**			**Measures of the suitability of the one-factor model**			
		**Parallel analysis**	**Velicer's MAP**	**Hull test**	**χ^2^ (df)**	**TLI**	**RMSEA**	**RMSR**
**One-dimensionality**								
**NA**	**Emotional lability**	2	2	2	1,100 (14)	0.439	0.320	0.170
**NA**	**Anxiousness**	2	1	2	640 (27)	0.755	0.176	0.080
**NA**	**Separation insecurity**	4	1	1	130 (14)	0.926	0.108	0.050
**NA**	**Submissiveness**	2	1	-	27 (2)	0.929	0.131	0.040
**NA**	**Hostility**	3	2	1	540 (35)	0.809	0.141	0.080
**NA**	**Perseveration**	4	1	2	370 (27)	0.815	0.133	0.080
**DET**	**Withdrawal**	2	1	1	190 (35)	0.955	0.078	0.030
**DET**	**Intimacy avoidance**	2	1	1	69 (9)	0.946	0.095	0.040
**DET**	**Anhedonia**	4	1	1	210 (20)	0.903	0.113	0.050
**DET**	**Depressivity**	3	1	2	1,090 (77)	0.876	0.134	0.050
**DET**	**Restricted Affectivity**	3	1	1	140 (14)	0.887	0.110	0.050
**DET**	**Suspiciousness**	2	1	1	140 (14)	0.763	0.111	0.080
**ANT**	**Manipulativeness**	2	1	-	74 (5)	0.930	0.137	0.040
**ANT**	**Deceitfulness**	4	1	1	500 (35)	0.862	0.135	0.060
**ANT**	**Grandiosity**	3	1	1	88 (9)	0.907	0.110	0.050
**ANT**	**Attention seeking**	2	1	2	350 (20)	0.880	0.150	0.070
**ANT**	**Callousness**	-	1	1	1,037 (77)	0.817	0.131	0.070
**DIS**	**Irresponsibility**	3	1	1	90 (14)	0.922	0.086	0.040
**DIS**	**Impulsivity**	1	1	1	31 (9)	0.985	0.058	0.020
**DIS**	**Distractibility**	2	1	1	220 (27)	0.933	0.099	0.040
**DIS**	**Risk Taking**	3	2	2	985.3 (77)	0.701	0.127	0.110
**DIS**	**Rigid perfectionism**	2	1	1	290 (35)	0.900	0.099	0.050
**PSY**	**Unusual beliefs & experiences**	4	1	1	280 (20)	0.827	0.132	0.070
**PSY**	**Eccentricity**	3	1	1	780.9 (65)	0.910	0.123	0.040
**PSY**	**Perceptual dysregulation**	5	1	1	631.2 (54)	0.804	0.121	0.070

NA, Negative Affect; DET, Detachment; ANT, Antagonism; DIS, Disinhibition; PSY, Psychoticism; RMSEA, Root Mean Square Error of Approximation; RMSR, Root Mean Square Residual; TLI, Tucker Lewis Index; X^2^, Chi-square.

##### Confirmatory factor analysis

The CFA results concerning the model proposed by Krueger et al. (4) showed that both Chi-square, Chi-square/df, and comparative goodness of fit indexes (CFI and TLI) are not satisfactory, and only the residual goodness of fit indexes (RMSEA and SRMR) were within acceptable parameters (Table 4). The factor loadings of the items in the reference facets (Supplementary material SM6) are below the expected, especially the Suspiciousness facet. As for the factor loadings of facets in the domains, most of them were adequate to the model tested, except the Disinhibition domain, which includes a facet with slightly lower loadings (<0.30: Rigid Perfectionism). Therefore, we used EFA to explore alternative models.

**Table 4:** PID-5 (paper-and-pencil) confirmatory and exploratory factor analysis of goodness of fit indexes (*N* = 730).

**Confirmatory factor analysis**			
**Indexes**	**Original model**		
**X** ^**2**^ **(df), p-value**	336372.826 (23835), <0.0001		
**X** ^**2**^ **/df**	14.11		
**SRMR**	0.082		
**RMSEA**	0.051		
**CFI**	0.511		
**TLI**	0.505		
**Exploratory factor analysis**			
**Indices**	**4 factors**	**5 factors**	**6 factors**
**χ^2^ (df), p-value**	747.290 (206), <0.0001	893.700 (185), <0.0001	581.010(165), <0.0001
**X** ^**2**^ **/df**	3.627	4.830	3.521
**TLI**	0.804	0.886	0.925
**RMSEA**	0.098	0.072	0.059
**RMSR**	0.040	0.030	0.020

CFI, Comparative Fit Index; df, Degrees of Freedom; IM, Modification gl Index; RMSEA, Root Mean Square Error of Approximation; SRMR, Standardized Root Mean Square Residuals; TLI, Tucker-Lewis Index; X^2^, Chi-Square test.

##### Exploratory factor analysis

The KMO index (0.923) and Bartlett's sphericity test (*p* < 0.001) showed the matrix factorability. The techniques used to retain the factors suggest the presence of four (Hull Test and Velicer's MAP) or six factors (Parallel Analysis). The goodness of fit indexes for each factor solution suggested and the five-factor model proposed by Krueger et al. (4) are presented in Table 4.

The joint analysis of goodness of fit indexes indicates that the five- and six-factor models are more suitable. However, considering the theoretical structure supporting the instrument and the factor loading of the facets in the domains, the five-factor model was chosen, which, compared to the original model proposed by Krueger et al. (4), presents some peculiarities in the composition of factors and interstitiality between some facets (Table 5 and Supplementary material SM7). Factor 1 was composed of the six original facets of Negative Affect, besides Suspiciousness (Detachment), Impulsivity, and Distractibility (Disinhibition). Additionally, it has a common factor loading with the other five facets belonging to the remaining domains. Factor 2 corresponds to the original grouping of five facets linked to Antagonism, while Factor 3 includes the original facets of Detachment, except Suspiciousness. Factor 4 was composed of only two original facets that composed the Disinhibition domain (Rigid Perfectionism and Irresponsibility), and Factor 5 includes the Psychoticism original facets in addition to Risk Taking, which originally belonged to Disinhibition.

**Table 5:** Factor loadings of PID-5 (paper-and-pencil) domains according to Exploratory Factor Analysis for the 5-factor model (*N* = 730).

**Facets (original domain)**	**Factor 1**	**Factor 2**	**Factor 3**	**Factor 4**	**Factor 5**
Emotional lability (NA)	**0.83**	−0.24	−0.29	0.11	0.23
Anxiousness (NA)	**0.83**	−0.10	0.01	0.22	−0.14
Separation insecurity (NA)	**0.69**	0.12	−0.18	0.13	−0.11
Submissiveness (NA)	**0.53**	0.20	0.00	−0.04	−0.20
Hostility (NA)	**0.31**	0.28	0.16	0.13	0.05
Perseveration (NA)	**0.54**	0.04	0.17	0.23	0.07
Withdrawal (DET)	0.09	−0.04	**0.82**	0.14	−0.08
Intimacy avoidance (DET)	−0.15	−0.09	**0.62**	0.02	0.07
Anhedonia (DET)	0.55	−0.15	**0.62**	−0.11	−0.17
Depressivity (DET)	0.67	−0.12	**0.40**	−0.15	−0.01
Restricted affectivity (DET)	−0.18	0.17	**0.69**	0.10	0.05
Suspiciousness (DET)	**0.36**	0.14	0.17	0.17	−0.02
Manipulativeness (ANT)	0.00	**0.87**	−0.10	0.05	0.03
Deceitfulness (ANT)	0.07	**0.82**	0.07	−0.13	−0.04
Grandiosity (ANT)	−0.15	**0.56**	0.02	0.40	0.13
Attention seeking (ANT)	0.33	**0.59**	−0.28	0.10	0.04
Callousness (ANT)	−0.13	**0.50**	0.46	−0.03	0.09
Irresponsibility (DIS)	0.30	0.37	0.13	**−0.33**	0.18
Impulsivity (DIS)	**0.53**	0.08	−0.15	−0.18	0.22
Distractibility (DIS)	**0.66**	0.04	0.08	−0.19	0.07
Risk Taking (DIS)	−0.23	0.27	−0.03	−0.15	**0.47**
Rigid perfectionism (DIS)	0.13	−0.04	0.11	**0.67**	0.02
Unusual beliefs & experiences (PSY)	0.12	−0.02	0.03	0.30	**0.59**
Eccentricity (PSY)	0.21	−0.02	0.34	0.08	**0.47**
Perceptual dysregulation (PSY)	0.49	−0.04	0.10	0.13	**0.45**

ANT, Antagonism; DET, Detachment; DIS, Disinhibition; NA, Negative Affect; PSY, Psychoticism; Bold, factor loadings above 0.30 were considered to assign the facets to domains.

##### Hierarchical structure

**Figure 1:**
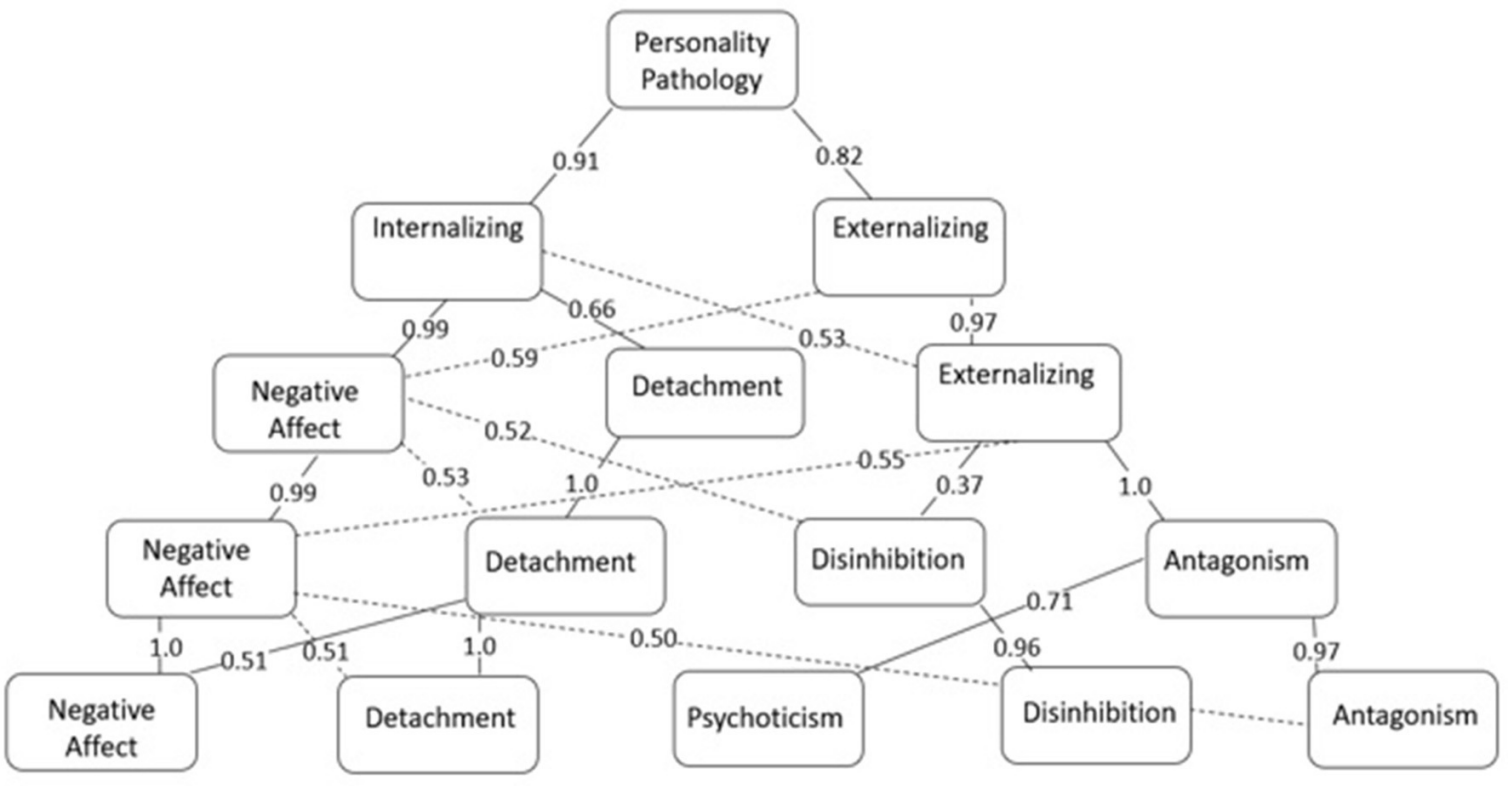
PID-5 (paper-and-pencil) five-level hierarchical structure (*N* = 730)—Path Coefficients between subordinate and superordinate factors.

The analysis of the PID-5 hierarchical structure (one to five factors) and path coefficients (>0.50) are presented in Figure 1 and Supplementary material SM8. The facets in the one-factor solution presented high factor loading (>0.30; except Risk Taking), indicating a general pathological personality factor predominantly characterized by Perceptual Dysregulation traits (0.80), Perseveration (0.76), and Depressivity (0.74).

In the lower level, this general factor is divided into two other levels that can be called Internalizing Symptoms (Anxiousness (0.97), Emotional Lability (0.81), and Depressivity (0.75), presented the highest factor loadings) and Externalizing Symptoms (Deceitfulness (0.85), Manipulativeness (0.83), and Callousness (0.81) presented the highest factor loadings).

In the third hierarchical level, the Externalizing Symptoms were kept in the structure – the facets related to Antagonism are those with the highest loadings: Manipulativeness (0.89), Deceitfulness (0.80), Attention Seeking (0.60), Callousness (0.58), and Grandiosity (0.55) –, and the Internalizing Symptoms were divided into other two domains, one of which is primarily represented by facets of the Negative Affect, e.g., Anxiousness (0.94), Emotional Lability (0.93), and Separation Insecurity (0.75), while the other is composed of the facets of the Detachment domain –Withdrawal (0.83), Restricted Affectivity (0.70), and Intimacy Avoidance (0.64).

In the fourth level, Negative Affect and Detachment kept the same structure. At the same time, Externalizing Symptoms were divided into two factors characterized by Antagonism traits: Manipulativeness (0.54), Deceitfulness (0.79), and Callousness (0.57) and Disinhibition (e.g., Rigid Perfectionism (0.72) and Unusual Beliefs & Experiences (0.30), and Irresponsibility (0.30).

The domains from the previous level were kept in the fifth and last level, and a new factor, called Psychoticism, emerged. The Negative Affect domain contains the facets with the highest loadings – Emotional Lability (0.83), Anxiousness (0.83), and Separation Insecurity (0.69) –, while the Withdrawal (0.83), Restricted Affectivity (0.62), and Intimacy Avoidance (0.62) facets from the Detachment domain presented the highest loadings. In turn, the Antagonism domain was mainly represented by the Manipulation (0.87), Deceitfulness (0.82), and Attention Seeking (0.59) facets, while Disinhibition was represented by Rigid Perfectionism (0.67), Deceitfulness (0.40), and Irresponsibility (-0.33). Finally, the new domain, Psychoticism, was more strongly represented by Unusual Beliefs & Experiences (0.59), Eccentricity (0.47), and Perceptual Dysregulation (0.45).

## Discussion

This study aimed to identify psychometric evidence of validity and reliability of the PID-5 Brazilian version (paper-and-pencil) in a population sample. Despite some divergences, especially regarding its internal structure, which we discuss below, the indexes generally indicate the instrument is appropriate.

The reliability, internal consistency, and temporal stability indexes proved to be adequate, similar to the studies addressing the original English version [α ≥ 0.72; (4)] and cross-cultural adaptations addressing general populations [α ≥ 0.68; *r* ≥ 0.56: (50–52)]. However, the Suspiciousness facet is an exception because its internal consistency indicators were below the expected (α ≤ 0.69), a finding already reported by other studies (11, 17, 19, 53).

The test-retest reliability indicators for Suspiciousness (ICC = 0.46) and Risk-Taking (ICC = 0.45) were also slightly lower than expected. A potential explanation concerns reverse-scored items, considering that these are the only facets with more than one item with this characteristic (Suspiciousness = 2 items; Risk Taking = 5 items). Additioanlly, reverse-scored items tend to present the lowest correlations with the total and individual temporal stability. This technique is widely used to identify response bias; however, Podsakoff et al. (54) noted that changing the response pattern might confuse respondents and compromise the instrument's psychometric qualities. Thus, Keeley et al. (55); Response Inconsistency Scale propose other strategies to minimize response bias, such as developing specific scales, which can be advantageous and more appropriate for this purpose without impacting the instruments' psychometric quality.

The Negative Affect domain and one of its facets (Anxiousness) obtained the highest scores. These scores indicate that nervousness, tension, and panic when facing stress, excessive worry, and various negative emotions were the pathological personality traits most frequently reported by the Brazilian population. Different studies have also found this pattern in general (11, 29, 56) and in clinical populations (11, 50).

On the other hand, the Antagonism domain, characterized by oppositional traits, exaggeration about oneself, and low empathy, and its Callousness facet, which highlights decreased empathy, lack of guilt, or consideration for others, obtained the lowest scores. Similar results were found in American (57), Spanish (19), and Hungarian community samples (11).

Interestingly, almost all items showed a floor effect; many answers were concentrated at the lowest levels, which may negatively impact the instrument's sensitivity and specificity. Considering it is a community sample, we suggest this aspect be explored in clinical samples to verify whether the instrument can discriminate differences and changes (responsiveness) (58). Note that the items with the lowest variability among answers (percentage of responses above 75% in category 0 “Very false or often false”) belong to the Callousness and Manipulativeness facets, suggesting a potential social desirability effect (59, 60). As for the ceiling effect (49), seven out of the 23 items with this effect were reverse-scored, suggesting that this characteristic led to response bias.

Regarding convergent validity, the results reveal strong correlations between the PID-5 and NEO-FFI domains and reinforce the theoretical expectation of an inverse association between normative and pathological personality traits (61, 62). Strong associations between Neuroticism and Negative Affect suggest that individuals with maladjustment and emotional instability experience a wide range of negative affect and emotions such as Insecurity, Hostility, and Anxiousness (1, 63). Likewise, correlations between the NEO-FFI Extroversion domain, which portrays sociability, assertiveness, and extroversion traits, were inversely proportional to the personality traits composing the PID-5 Detachment domain, which depicts social avoidance experience, including withdrawal in interpersonal interactions, affective experience, and expression (1, 63). Negative associations between Antagonism and Agreeableness indicate the presence of oppositional, egocentric, manipulative, and little empathic traits, in contrast to traits that express the ability to put oneself in someone else's shoes and feelings of compassion and complacency. The same was found between Disinhibition, characterized by Impulsivity, Irresponsibility, and Risk-Taking, among others, and Conscientiousness, which reflects impulse control, the ability to plan, organize and perform tasks (1, 64, 65).

The relationship between Psychoticism and Openness to Experience is not consensus in the literature (66–68), which might explain the weak and negative correlations found in this study between these domains. Krueger et al. (4) considered that Openness to Experience was unrelated to personality disorders. However, Pocnet et al. (69) considered that the PID-5 Psychoticism domain does not fit as an FFM variant, being only an associate construct, which according to Krueger and Tackett (70), is indispensable to characterize the Schizotypal personality disorder, presenting incremental value as a separate and additional spectrum to the four-factor integrative personality model (71). In turn, Chmielewski et al. (72) suggest that this domain be considered a sixth factor because it is different from the pattern of the other domains involving the FFM.

As for the indicators related to the PID-5 internal structure, it is worth noting that the initial analysis related to the facets' unidimensionality, as proposed by Krueger et al. (4), cannot be tested for ten of them, as these seem to be better explained by a two-dimension model. This divergence from the theoretical model has already been evidenced in other studies, especially concerning the Emotional Lability and Hostility facets, which more usually replicate a two-dimension structure (11, 19, 20, 51, 56, 73).

Zimmerman et al. (73) consider that Hostility can be composed of two factors: one involving negative emotional states and the other involving a more behavioral and antagonistic aspect, which, they argue, could also explain why this facet's factor loadings oscillate between Negative Affect and Antagonism. Likewise, according to Gutiérrez et al. (19), two components representing items linked to strong, mutable, and unstable emotions and items that characterize a tendency to emotional susceptibility seem to explain Emotional Lability better. In general, data found in this study regarding these facets follow this direction and reinforce these new structural propositions.

As in the study by Riegel et al. (20), the Depressivity facet seems to be better explained by a dimension characterized by feelings of worthlessness, guilt, and suicidal thoughts and desire. Regarding the Suspiciousness and Risk-Taking facets, as noted by Riegel et al. (20), there seems to be a bias related to the instrument's response pattern, in which a factor represents items with direct scores and the other with reverse-scored items.

As for the Perseveration facet, 56 considered it a two-dimension facet as they found difficulties interpreting the differences between the factors, considering these difficulties to result from potential social desirability. However, in this study, persistent dysfunctional behaviors characterized one dimension, and the persistence of fixation in tasks characterized the other.

As far as we know, this is the first time that Anxiousness, Attention Seeking, Unusual Beliefs & Experiences, and Perceptual Dysregulation adjust better to a two-dimension structure. Anxiousness seems to be better explained by a dimension linked to worry focused on past and future experiences and a dimension linked to pervasive experiences of Anxiousness and worry. Attention Seeking includes items linked to Attention Seeking itself and a search for Admiration, which seems to include a specific dimension. Unusual Beliefs & Experiences seem to be composed of a dimension characterized by uncommon cognitive and sensory experiences and a dimension composed of idiosyncratic skills. Finally, the Perceptual Dysregulation facet seems composed of a component in which these experiences are linked to a perception of oneself and others and a dimension linked to the perception of the external environment.

These findings reinforce previous statements of Riegel et al. (20), according to which the issue of an instrument's unidimensionality has been a problem since the original study by Krueger et al. (4). For this reason, Riegel et al. (20) opted for performing an EFA instead of a CFA because they considered the facets' structure premature, emphasizing the high risk of interstitiality between them and consequently reflecting a lack of unidimensionality. In addition, they proposed that the 60 items presenting low factor loadings in the reference facet or substantial residual covariance with other items be removed from the instrument to improve the clarity of the facet concept.

Krueger and Markon (61) defend that a personality structure is complex and composed of multiple naturally interstitial traits. They argue that eliminating indicators because they do not exhibit a one-dimension structure, even if psychometrically convenient, would increase the risk of creating an incomplete instrument that does not represent a personality or psychopathology. Likewise, considering that these indicators belong to a simple structure favors a potential distortion in the constructs' nature.

The complexity of the theoretical model proposed by Krueger et al. (4) is also reflected at the level of facets/domains because it was impossible to replicate the original model using CFA, considering that the goodness of fit indexes was not satisfactory. Riegel et al. (20) reported this fact in a study addressing population and clinical samples in the Czech Republic (RMSEA = 0.108; CFI = 0.762), although Al-Attiyah et al. (29) replicated the original structure in an Arab college sample (RMSEA = 0.05; CFI = 0.97).

Many other studies focused on the EFA of PID-5 internal structure (17, 18, 20, 25, 28, 67, 73–78) and reported models that partially diverged from the original model, especially regarding the composition of the Negative Affect domain. Many facets that initially belonged to other domains presented a higher factor loading in this domain, expanding it in the instrument's composition.

Our findings go in this direction, as the five-factor model, similar to the original model, appeared to be the best. Divergences concern the Negative Affect domain, which grouped a more significant number of facets (Suspiciousness, Impulsivity, and Distractibility), and the Disinhibition domain, which was composed of only two of its original facets (Irresponsibility, Rigid Perfectionism). Regarding Negative Affect, previous studies have repeatedly shown the Suspiciousness' (Detachment) high factor loading in this domain (17, 18, 20, 28, 76, 78). On the other hand, Lotfi et al. (78) had already reported an association between the Impulsivity and Distractibility facets, which were initially linked to Disinhibition and Negative Affect. Lotfi et al. (78) consider that its link to Negative Affect is easily explained because Impulsivity corresponds to one of the Neuroticism's facets (79). However, difficulty concentrating and maintaining goal-oriented behaviors are common when experiencing negative emotions, which could explain the association of Distractibility to this domain, characterizing one of the behavioral manifestations of this affective experience. We believe this rationale also explains the high loading of Suspiciousness into Negative Affect, which would compose one of the behavioral and interrelational manifestations associated with negative affect.

As for the Disinhibition domain, some studies (20, 73, 74) fail to fully replicate it with its original facets loading more strongly in different domains and different ways. Hence, the presence of latent domains is suggested [Compulsivity and Impulsivity; (20)]. From this perspective, this domain is more strongly represented in the Brazilian sample by compulsiveness latent traits, mainly expressed by Rigid Perfectionism (absence), considering that Impulsivity latent traits (represented by Impulsivity and Distractibility), characterizing an emotionally unstable affect, presented a more significant association with Negative Affect.

Roskam et al. (28) also found an association between Risk-Taking to the Psychoticism domain. A potential explanation for this grouping is that this facet integrates the list of unusual behaviors that ignore reality; in this case, by denying risks and dangers associated with the behavior.

Additionally, the high frequency of interstitiality between the facets draws attention, which Watters and Bagby (80) report in their meta-analysis. Even though these findings are expected, given the complexity of personality-associated theoretical models, they indicate a need to improve studies addressing the PID-5 discriminative ability. To decrease the problem associated with interstitiality, Krueger et al. (81) proposed an alternative method to correct the instrument, in which the three facets with the highest factor loadings in each domain would result in real markers.

Our findings partially support these propositions because three of the 15 facets selected still presented interstitiality in our sample: Anhedonia (Detachment), Impulsivity, and Distractibility (Disinhibition) with Negative Affect. Therefore, there is no evidence supporting the use of this algorithm in Brazil, and these aspects need to be extensively investigated in the future. Furthermore, Krueger et al. (81) also highlight the limitations of disregarding the other facets at the risk of damaging the original model. Finally, the results reported by Watters, Sellbom, and Bagby (82) indicate different results when applying different scoring methods for the domains, with implications for interpreting the results.

Analyzes were also conducted to verify whether PID-5 could replicate the hierarchical structure of personality, as proposed by Widiger and Simonsen (83), in which the top of the structure represents broad dimensions of personality associated with common mental disorders, down to the lowest level, characterized by specific trait scales. Studies conducted by Aboul Ata et al. (17), Lotfi et al. (78), Gutierrez et al. (19), and Roskam et al. (28) replicated this model in Egyptian, Iranian, and French community samples, as we did in Brazil. The findings are generally aligned with pre-established personality and psychopathology models. Achenbach (84) and Krueger (85) noted that internalizing and externalizing characteristics predominate at the two-factor level. At the third level, the grouping is similar to the propositions associated with the study of temperament (86), in which three dimensions are highlighted: Extraversion/urgency (represented by Detachment in the current structure), Effortful Control (represented by Disinhibition), and Negative Affect. At the fourth level, Livesley et al. (87) proposed that the groupings can be represented by Emotional Dysregulation traits (which would correspond to facets linked to Negative Affect), Dissocial Behavior (linked to Antagonism), Inhibition (linked to Detachment), and Compulsiveness (basically represented by a lack of Rigid Perfectionism in the Disinhibition domain). Finally, at the fifth level, we have a model that is close to the maladaptive variant of the Big Five Model (88), as proposed by the DSM−5 (1), or the Personality Psychopathology model – Five [PSY−5; (89): Aggressive, Psychotic, Constraint, Negative Emotion, Extraversion/Positive Emotion].

The findings indicate that the instrument presents favorable psychometric indicators enabling its use in the Brazilian context. However, these findings should not be generalized, considering that the sample was predominantly composed of working female, young adults with a high educational level.

The main critical aspects concern: a) the impact of reversed-score items in the reliability indicators; b) the floor effect found in a considerable number of items, suggesting social desirability; c) the lack of unidimensionality of some facets; and d) the presence of interstitiality and specificities related to the instrument's internal structure, which did not fully replicate the original model. On the other hand, adequacy to the hierarchical model indicates it is aligned with the personality theoretical models.

Together with extensive psychometric literature on PID-5, these results reinforce the need to investigate these topics further, which concern restrictions common among the various cross-cultural adaptations and samples. Therefore, it seems appropriate to a) revise the PID-5 internal structure, considering the possibility of removing items, based on a specific analysis of the Item Response Theory (IRT), and b) assess its discriminative ability, especially to operationalize the DSM-5 dimensional personality model.

## Data availability statement

The raw data supporting the conclusions of this article will be made available by the authors, without undue reservation.

## Ethics statement

The studies involving human participants were reviewed and approved by Comitê de Ética do Hospital das Clínicas da Faculdade de Medicina de Ribeirão Preto (process n° 4058/2018). The patients/participants provided their written informed consent to participate in this study.

## Author contributions

Conceptualization and methodology: AB-F and FO. Formal analysis and writing—original draft: AB-F. Supervision and writing—review and editing: FO. Both authors contributed to the article and approved the submitted version.

## Funding

This work was funded by the São Paulo Research Foundation (FAPESP - Process No. 2019/27022-0), Coordination for the Improvement of Higher Education Personnel (CAPES), and Brazilian National Council for Scientific and Technological Development, Productivity Research Fellows, CNPq- Process No. 302601/2019-8.

## Conflict of interest

The authors declare that the research was conducted in the absence of any commercial or financial relationships that could be construed as a potential conflict of interest.

## Publisher's note

All claims expressed in this article are solely those of the authors and do not necessarily represent those of their affiliated organizations, or those of the publisher, the editors and the reviewers. Any product that may be evaluated in this article, or claim that may be made by its manufacturer, is not guaranteed or endorsed by the publisher.
