# Supplementary material for: Psychometric study of the brazilian version of the personality inventory for DSM-5–paper-and-pencil version
Source: Front Psychiatry. 2022 Sep 13;13:976831. doi: 10.3389/fpsyt.2022.976831 (PMC9513057; doi:10.3389/fpsyt.2022.976831)
Supplement: Supplementary file 1 [file Data_Sheet_1.docx]

Supplementary Material SM1: Sociodemographic characterization of the sample concerning PID-5 (paper-and-pencil) (N=730) and comparison with the sample excluded due to missing data (N= 380)

| **Variables** | **Final sample** | | **Sample excluded due to missing data** | | **p value** |
| --- | --- | --- | --- | --- | --- |
|  | **N** | **%** | **N** | **%** |  |
| **Sex** |  |  |  |  | 0.001* |
| Male | 235 | 32.20 | 86 | 22.6 |  |
| Female | 495 | 67.80 | 294 | 77.4 |  |
| **Age (Mean; SD)** | 33.84 (15.15) |  | 36.1 (17.00) |  | 0.030* |
| **Children** |  |  |  |  | 0.028* |
| No | 453 | 62.10 | 208 | 54.7 |  |
| Yes | 265 | 36.30 | 162 | 42.6 |  |
| Not reported | 12 | 1.60 | 10 | 2.6 |  |
| **Marital Status** |  |  |  |  | 0.406 |
| No partner/spouse | 493 | 68.50 | 251 | 66.0 |  |
| Partner/spouse | 227 | 31.10 | 125 | 32.9 |  |
| Not reported | 4 | 0.4 | 4 | 1.1 |  |
| **Education** |  |  |  |  | 0.001* |
| Up to 12 years of schooling | 223 | 30.50 | 140 | 39.5 |  |
| >12 years of schooling | 507 | 69.50 | 222 | 58.4 |  |
| Not reported | 0 | 0.00 | 8 | 2.1 |  |
| **Employment** |  |  |  |  |  |
| Employed^(1)^ | 590 | 80.80 | 170 | 44.7 |  |
| Not employed | 130 | 17.80 | 198 | 52.2 |  |
| Not reported | 10 | 1.40 | 12 | 3.2 |  |
| **Health Problems** |  |  |  |  | 0.381 |
| No | 570 | 78.10 | 284 | 74.7 |  |
| Yes, in the past | 54 | 7.40 | 33 | 8.7 |  |
| Yes, at the moment | 101 | 13.80 | 62 | 16.3 |  |
| Not reported | 5 | 0.70 | 1 | 0.3 |  |
| **Psychiatric diagnosis** |  |  |  |  | 0.426 |
| No | 624 | 85.50 | 334 | 87.9 |  |
| Yes | 100 | 13.70 | 46 | 12.1 |  |
| Not reported | 6 | 0.80 | 0 | 0.00 |  |
| **Psychotherapy** |  |  |  |  | 0.543 |
| No | 460 | 64.00 | 251 | 66.1 |  |
| Yes, in the past | 166 | 22.70 | 76 | 20.0 |  |
| Yes, at the moment | 96 | 12.70 | 48 | 12.6 |  |
| Not reported | 5 | 0.6 | 5 | 1.3 |  |
| **Regular use of medication** |  |  |  |  | 0.333 |
| No | 467 | 64.00 | 231 | 60.8 |  |
| Yes | 259 | 35.50 | 143 | 37.6 |  |
| Not reported | 4 | 0.50 | 6 | 1.6 |  |
| **Tobacco consumption** |  |  |  |  | 0.018* |
| No | 623 | 85.30 | 310 | 81.6 |  |
| Yes, in the past | 43 | 5.90 | 40 | 10.5 |  |
| Yes, at the moment | 54 | 7.40 | 24 | 6.3 |  |
| Not reported | 16 | 1.40 | 6 | 1.6 |  |
| **Alcohol consumption** |  |  |  |  | 0.334 |
| No | 316 | 43.30 | 165 | 43.4 |  |
| Yes, in the past | 37 | 5.20 | 28 | 7.4 |  |
| Yes, at the moment | 370 | 50.70 | 184 | 48.4 |  |
| Not reported | 6 | 0.80 | 3 | 0.8 |  |
| **Troubles with the law** |  |  |  |  | 0.935 |
| No | 713 | 97.70 | 373 | 98.2 |  |
| Yes | 11 | 1.50 | 6 | 1.6 |  |
| Not reported | 6 | 0.80 | 1 | 0.3 |  |
| **Response Inconsistency Scale (Mean; SD)** | 9.20 (7.37) | | 9.59 (4.42) | | 0.16 |
| **Response Inconsistency Scale (Cut-off >17) ^(a)^** | 4.5% | | 4.7% | |  |

¹= Employed individuals or students; SD = Standard Deviation; ^(a)^ Calculated according to the recommendations of Keeley, J. W., Webb, C., Peterson, D., Roussin, L., & Flanagan, E. H. (2016). Development of a response inconsistency scale for the personality inventory for DSM–5. Journal of Personality Assessment, 98(4), 351-359 and Bagby, M.R., & Sellbom, M. (2018). The Validity and Clinical Utility of the Personality Inventory for DSM–5 Response Inconsistency Scale, Journal of Personality. Assessment, 398-405.

Supplementary Material SM2 – Raw Scores, Distribution Measures, Item-Total correlation, and PID-5 (paper-and-pencil) Test-Retest Reliability (N=730)

| **Item** | **Raw Scores** | | **Distribution Measure** | | | | **Item-Total Correlation** | **T/R** |
| --- | --- | --- | --- | --- | --- | --- | --- | --- |
|  | **Mean** | **SD** | **Ass** | **(SE)** | **Kurt** | **(SE)** |  | **ICC (95%)** |
| **P1** | 1.22 | 0.98 | 0.12 | 0.09 | -1.15 | 0.18 | 0.45 | 0.55 (0.35-0.71) |
| **P2** | 0.39 | 0.77 | 1.91 | 0.09 | 2.67 | 0.18 | 0.26 | 0.32 (0.07-0.53) |
| **P3** | 0.35 | 0.67 | 2.03 | 0.09 | 3.69 | 0.18 | 0.33 | 0.28 (0.03-0.50) |
| **P4** | 0.97 | 0.93 | 0.55 | 0.09 | -0.70 | 0.18 | 0.38 | 0.48 (0.25-0.66) |
| **P5** | 0.97 | 1.05 | 0.65 | 0.09 | -0.89 | 0.18 | 0.60 | 0.38 (0.14-0.58) |
| **P6** | 1.16 | 1.00 | 0.39 | 0.09 | -0.95 | 0.18 | 0.46 | 0.43 (0.16-0.61) |
| **P7** | 0.93 | 0.94 | 0.66 | 0.09 | -0.60 | 0.18 | 0.02 | 0.06 (-0.50-0.31) |
| **P8** | 0.99 | 1.04 | 0.59 | 0.09 | -0.95 | 0.18 | 0.38 | 0.08 (0.07-0.88) |
| **P9** | 1.13 | 0.94 | 0.32 | 0.09 | -0.91 | 0.18 | 0.34 | 0.14 (-0.50-0.38) |
| **P10** | 0.88 | 0.95 | 0.72 | 0.09 | -0.61 | 0.18 | 0.46 | 0.49 (0.27-0.66) |
| **P11** | 0.13 | 0.53 | 4.47 | 0.09 | 19.74 | 0.18 | 0.21 | 0.00 (-0.25-0.26) |
| **P12** | 1.01 | 1.08 | 0.66 | 0.09 | -0.92 | 0.18 | 0.36 | 0.53 (0.31-0.69) |
| **P13** | 0.44 | 0.72 | 1.56 | 0.09 | 1.62 | 0.18 | 0.40 | 0.66 (0.49-0.78) |
| **P14** | 0.58 | 0.80 | 1.18 | 0.09 | 0.46 | 0.18 | 0.42 | 0.34 (0.10-0.55) |
| **P15** | 0.78 | 0.87 | 0.78 | 0.09 | -0.41 | 0.18 | 0.38 | 0.36 (0.12-0.56) |
| **P16** | 0.75 | 0.85 | 0.86 | 0.09 | -0.16 | 0.18 | 0.42 | 0.22 (-0.48-0.44) |
| **P17** | 0.67 | 0.83 | 1.06 | 0.09 | 0.31 | 0.18 | 0.47 | 0.51 (0.30-0.68) |
| **P18** | 1.29 | 1.04 | 0.22 | 0.09 | -1.15 | 0.18 | 0.51 | 0.51 (0.30-0.68) |
| **P19** | 0.28 | 0.63 | 2.31 | 0.09 | 4.76 | 0.18 | 0.36 | 0.46 (0.23-0.64) |
| **P20** | 1.35 | 1.09 | 0.15 | 0.09 | -1.28 | 0.18 | 0.55 | 0.42 (0.19-0.61) |
| **P21** | 0.77 | 0.93 | 0.95 | 0.09 | -0.19 | 0.18 | 0.58 | 0.50 (0.29-0.67) |
| **P22** | 0.85 | 0.84 | 0.63 | 0.09 | -0.43 | 0.18 | 0.47 | 0.59 (0.39-0.73) |
| **P23** | 0.71 | 0.92 | 1.09 | 0.09 | 0.16 | 0.18 | 0.54 | 0.42 (0.18-0.60) |
| **P24** | 0.62 | 0.85 | 1.24 | 0.09 | 0.62 | 0.18 | 0.59 | 0.57 (0.37-0.72) |
| **P25** | 0.55 | 0.85 | 1.42 | 0.09 | 1.03 | 0.18 | 0.57 | 0.60 (0.41-0.44) |
| **P26** | 0.8 | 0.98 | 0.95 | 0.09 | -0.27 | 0.18 | 0.46 | 0.64 (0.46-0.77) |
| **P27** | 0.78 | 0.99 | 0.98 | 0.09 | -0.28 | 0.18 | 0.62 | 0.63 (0.44-0.76) |
| **P28** | 0.97 | 0.95 | 0.67 | 0.09 | -0.50 | 0.18 | 0.46 | 0.59 (0.40-0.74) |
| **P29** | 0.69 | 0.83 | 0.98 | 0.09 | 0.13 | 0.18 | 0.48 | 0.58 (0.38-0.72) |
| **P30R** | 1.21 | 0.96 | 0.39 | 0.09 | -0.79 | 0.18 | 0.19 | 0.32 (0.08-0.53) |
| **P31** | 0.27 | 0.60 | 2.44 | 0.09 | 6.04 | 0.18 | 0.40 | 0.40 (0.17-0.60) |
| **P32** | 1.02 | 1.03 | 0.56 | 0.09 | -0.93 | 0.18 | 0.46 | 0.44 (0.21-0.62) |
| **P33** | 0.77 | 0.96 | 0.99 | 0.09 | -0.16 | 0.18 | 0.64 | 0.68 (0.52-0.80) |
| **P34** | 1.06 | 1.02 | 0.45 | 0.09 | -1.05 | 0.18 | 0.24 | 0.11 (-0.15-0.35) |
| **P35R** | 1.75 | 1.12 | -0.34 | 0.09 | -1.26 | 0.18 | -0.07 | 0.27 (0.02-0.49) |
| **P36** | 0.51 | 0.85 | 1.59 | 0.09 | 1.58 | 0.18 | 0.43 | 0.80 (0.68-0.87) |
| **P37** | 0.20 | 0.58 | 3.24 | 0.09 | 10.29 | 0.18 | 0.34 | 0.58 (0.39-0.73) |
| **P38** | 1.26 | 0.99 | 0.27 | 0.09 | -0.97 | 0.18 | 0.47 | 0.31 (0.06-0.53) |
| **P39** | 0.36 | 0.71 | 2.13 | 0.09 | 4.03 | 0.18 | 0.32 | 0.24 (-0.48-0.46) |
| **P40** | 0.47 | 0.85 | 1.80 | 0.09 | 2.16 | 0.18 | 0.36 | 0.57 (0.37-0.72) |
| **P41** | 0.29 | 0.64 | 2.40 | 0.09 | 5.49 | 0.18 | 0.40 | 0.49 (0.22-0.66) |
| **P42** | 0.50 | 0.79 | 1.54 | 0.09 | 1.61 | 0.18 | 0.36 | 0.49 (0.26-0.66) |
| **P43** | 0.64 | 0.84 | 1.13 | 0.09 | 0.33 | 0.18 | 0.46 | 0.34 (0.10-0.58) |
| **P44** | 0.29 | 0.64 | 2.32 | 0.09 | 4.85 | 0.18 | 0.37 | 0.22 (-0.04-0.45) |
| **P45** | 0.89 | 0.92 | 0.71 | 0.09 | -0.46 | 0.18 | 0.35 | 0.47 (0.24-0.65) |
| **P46** | 0.92 | 0.96 | 0.75 | 0.09 | -0.46 | 0.18 | 0.32 | 0.70 (0.55-0.81) |
| **P47** | 0.88 | 0.97 | 0.76 | 0.09 | -0.56 | 0.18 | 0.34 | 0.57 (0.37-0.72) |
| **P48** | 0.49 | 0.76 | 1.53 | 0.09 | 1.66 | 0.18 | 0.35 | 0.10 (-0.15-0.35) |
| **P49** | 1.40 | 1.04 | 0.05 | 0.09 | -1.19 | 0.18 | 0.18 | 0.40 (0.16-0.59) |
| **P50** | 1.11 | 1.03 | 0.42 | 0.09 | -1.03 | 0.18 | 0.39 | 0.58 (0.38-0.73) |
| **P51** | 1.20 | 1.03 | 0.33 | 0.09 | -1.08 | 0.18 | 0.32 | 0.48 (0.26-0.66) |
| **P52** | 0.77 | 0.93 | 0.95 | 0.09 | -0.17 | 0.18 | 0.58 | 0.22 (-0.04-0.45) |
| **P53** | 0.24 | 0.58 | 2.64 | 0.09 | 6.94 | 0.18 | 0.44 | 0.74 (0.58-0.84) |
| **P54** | 0.23 | 0.62 | 2.99 | 0.09 | 8.72 | 0.18 | 0.34 | 0.70 (0.54-0.81) |
| **P55** | 0.45 | 0.76 | 1.66 | 0.09 | 2.02 | 0.18 | 0.57 | 0.18 (-0.07-0.41) |
| **P56** | 0.31 | 0.63 | 2.17 | 0.09 | 4.49 | 0.18 | 0.39 | 0.58 (0.38-0.72) |
| **P57** | 0.28 | 0.69 | 2.66 | 0.09 | 6.37 | 0.18 | 0.33 | 0.35 (0.10-0.55) |
| **P58R** | 0.82 | 0.91 | 0.86 | 0.09 | -0.22 | 0.18 | 0.20 | 0.03 (-0.24-0.28) |
| **P59** | 0.86 | 1.04 | 0.85 | 0.09 | -0.60 | 0.18 | 0.43 | 0.27 (0.01-0.49) |
| **P60** | 0.71 | 0.80 | 0.88 | 0.09 | 0.04 | 0.18 | 0.49 | 0.32 (0.44-0.76) |
| **P61** | 1.01 | 1.03 | 0.60 | 0.09 | -0.89 | 0.18 | 0.61 | 0.58 (0.38-0.73) |
| **P62** | 0.99 | 1.02 | 0.61 | 0.09 | -0.84 | 0.18 | 0.49 | 0.30 (0.05-0.52) |
| **P63** | 0.72 | 0.79 | 0.82 | 0.09 | -0.12 | 0.18 | 0.35 | 0.68 (0.51-0.80) |
| **P64** | 0.43 | 0.77 | 1.80 | 0.09 | 2.43 | 0.18 | 0.39 | 0.58 (0.38-0.73) |
| **P65** | 0.89 | 0.97 | 0.66 | 0.09 | -0.79 | 0.18 | 0.28 | 0.51 (0.29-0.67) |
| **P66** | 0.48 | 0.86 | 1.73 | 0.09 | 1.92 | 0.18 | 0.51 | 0.41 (0.17-0.60) |
| **P67** | 0.53 | 0.77 | 1.28 | 0.09 | 0.77 | 0.18 | 0.28 | 0.51 (0.29-0.67) |
| **P68** | 0.70 | 0.85 | 0.93 | 0.09 | -0.13 | 0.18 | 0.54 | 0.44 (0.21-0.62) |
| **P69** | 1.10 | 0.93 | 0.33 | 0.09 | -0.90 | 0.18 | 0.28 | 0.41 (0.17-0.60) |
| **P70** | 0.55 | 0.82 | 1.34 | 0.09 | 0.84 | 0.18 | 0.58 | 0.68 (0.51-0.80) |
| **P71** | 0.55 | 0.83 | 1.33 | 0.09 | 0.74 | 0.18 | 0.63 | 0.57 (0.37-0.72) |
| **P72** | 0.25 | 0.63 | 2.63 | 0.09 | 6.47 | 0.18 | 0.40 | 0.84 (0.75-0.90) |
| **P73** | 0.22 | 0.54 | 2.61 | 0.09 | 6.86 | 0.18 | 0.40 | 0.72 (0.57-0.82) |
| **P74** | 0.54 | 0.79 | 1.26 | 0.09 | 0.56 | 0.18 | 0.45 | 0.51 (0.29-0.67) |
| **P75** | 0.73 | 0.85 | 0.90 | 0.09 | -0.13 | 0.18 | 0.45 | 0.55 (0.34-0.70) |
| **P76** | 0.52 | 0.81 | 1.40 | 0.09 | 1.01 | 0.18 | 0.45 | 0.67 (0.51-0.79) |
| **P77** | 0.36 | 0.68 | 1.84 | 0.09 | 2.52 | 0.18 | 0.32 | 0.35 (0.10-0.55) |
| **P78** | 0.71 | 0.84 | 0.90 | 0.09 | -0.15 | 0.18 | 0.43 | 0.29 (0.04-0.51) |
| **P79** | 1.47 | 1.06 | 0.01 | 0.09 | -1.22 | 0.18 | 0.44 | 0.27 (0.02-0.49) |
| **P80** | 0.87 | 0.91 | 0.66 | 0.09 | -0.62 | 0.18 | 0.52 | 0.23 (-0.02-0.47) |
| **P81** | 0.23 | 0.63 | 2.91 | 0.09 | 7.86 | 0.18 | 0.49 | 0.27 (0.01-0.49) |
| **P82** | 0.65 | 0.85 | 1.02 | 0.09 | -0.01 | 0.18 | 0.59 | 0.65 (0.48-0.78) |
| **P83** | 0.96 | 0.99 | 0.65 | 0.09 | -0.71 | 0.18 | 0.62 | 0.51 (0.29-0.68) |
| **P84** | 0.43 | 0.80 | 1.75 | 0.09 | 2.00 | 0.18 | 0.27 | 0.45 (0.22-0.63) |
| **P85** | 0.68 | 0.85 | 1.12 | 0.09 | 0.51 | 0.18 | 0.45 | 0.31 (0.06-0.52) |
| **P86** | 0.95 | 1.03 | 0.70 | 0.09 | -0.78 | 0.18 | 0.47 | 0.53 (0.32-0.69) |
| **P87R** | 1.76 | 1.06 | -0.35 | 0.09 | -1.11 | 0.18 | -0.11 | 0.25 (-0.09-0.47) |
| **P88** | 0.76 | 0.88 | 0.92 | 0.09 | -0.05 | 0.18 | 0.45 | 0.61 (0.43-0.75) |
| **P89** | 0.51 | 0.88 | 1.67 | 0.09 | 1.69 | 0.18 | 0.30 | 0.68 (0.52-0.80) |
| **P90R** | 1.01 | 1.19 | 0.61 | 0.09 | -1.25 | 0.18 | 0.19 | 0.20 (-0.06-0.44) |
| **P91** | 1.01 | 1.01 | 0.56 | 0.09 | -0.91 | 0.18 | 0.25 | 0.38 (0.14-0.58) |
| **P92** | 1.16 | 1.02 | 0.34 | 0.09 | -1.08 | 0.18 | 0.38 | 0.52 (0.31-0.68) |
| **P93** | 1.05 | 1.07 | 0.56 | 0.09 | -1.01 | 0.18 | 0.54 | 0.41 (0.17-0.60) |
| **P94** | 0.83 | 0.99 | 0.84 | 0.09 | -0.57 | 0.18 | 0.43 | 0.57 (0.36-0.72) |
| **P95** | 1.12 | 1.03 | 0.44 | 0.09 | -1.00 | 0.18 | 0.55 | 0.69 (0.52-0.80) |
| **P96R** | 2.37 | 0.88 | -1.23 | 0.09 | 0.48 | 0.18 | -0.09 | 0.07 ( -0.19-0.32) |
| **P97R** | 0.94 | 1.01 | 0.80 | 0.09 | -0.50 | 0.18 | 0.10 | 0.36 (0.11-0.56) |
| **P98R** | 0.70 | 0.86 | 1.02 | 0.09 | 0.15 | 0.18 | 0.12 | 0.06 (-0.20-0.30) |
| **P99** | 0.75 | 1.04 | 1.02 | 0.09 | -0.42 | 0.18 | 0.32 | 0.45 (0.21-0.63) |
| **P100** | 0.71 | 0.92 | 1.06 | 0.09 | 0.04 | 0.18 | 0.56 | 0.58 (0.38-0.73) |
| **P101** | 1.04 | 1.03 | 0.54 | 0.09 | -0.95 | 0.18 | 0.49 | 0.45 (0.22-0.64) |
| **P102** | 1.82 | 1.01 | -0.40 | 0.09 | -0.97 | 0.18 | 0.05 | 0.44 (0.19-0.62) |
| **P103** | 1.36 | 1.07 | 0.13 | 0.09 | -1.25 | 0.18 | 0.43 | 0.48 (0.25-0.65) |
| **P104** | 0.76 | 0.96 | 1.00 | 0.09 | -0.18 | 0.18 | 0.59 | 0.67 (0.51-0.79) |
| **P105** | 0.90 | 0.91 | 0.61 | 0.09 | -0.68 | 0.18 | 0.45 | 0.49 (0.27-0.66) |
| **P106** | 0.53 | 0.90 | 1.55 | 0.09 | 1.20 | 0.18 | 0.33 | 0.39 (0.16-0.59) |
| **P107** | 0.82 | 0.90 | 0.70 | 0.09 | -0.62 | 0.18 | 0.40 | 0.36 (0.12-0.57) |
| **P108** | 0.42 | 0.79 | 1.92 | 0.09 | 2.81 | 0.18 | 0.22 | 0.44 (0.21-0.62) |
| **P109** | 1.83 | 0.98 | -0.36 | 0.09 | -0.91 | 0.18 | 0.42 | 0.62 (0.43-0.75) |
| **P110** | 1.73 | 1.04 | -0.32 | 0.09 | -1.08 | 0.18 | 0.41 | 0.56 (0.36-0.71) |
| **P111** | 0.86 | 0.99 | 0.75 | 0.09 | -0.68 | 0.18 | 0.31 | 0.59 (0.39-0.73) |
| **P112** | 1.33 | 0.93 | 0.01 | 0.09 | -0.95 | 0.18 | 0.27 | 0.46 (0.23-0.64) |
| **P113** | 0.57 | 0.79 | 1.21 | 0.09 | 0.60 | 0.18 | 0.35 | 0.22 (-0.04-0.45) |
| **P114** | 0.43 | 0.76 | 1.73 | 0.09 | 2.14 | 0.18 | 0.37 | 0.54 (0.33-0.70) |
| **P115** | 0.92 | 1.01 | 0.73 | 0.09 | -0.67 | 0.18 | 0.22 | 0.59 (0.39-0.73) |
| **P116** | 0.47 | 0.74 | 1.57 | 0.09 | 1.82 | 0.18 | 0.47 | 0.45 (0.22-0.63) |
| **P117** | 1.63 | 1.04 | -0.14 | 0.09 | -1.14 | 0.18 | 0.27 | 0.41 (0.18-0.60) |
| **P118** | 1.08 | 1.00 | 0.45 | 0.09 | -0.96 | 0.18 | 0.51 | 0.43 (0.20-0.62) |
| **P119** | 0.27 | 0.68 | 2.71 | 0.09 | 6.73 | 0.18 | 0.45 | 0.35 (0.11-0.56) |
| **P120** | 0.73 | 0.97 | 1.07 | 0.09 | -0.09 | 0.18 | 0.21 | 0.51 (0.29-0.67) |
| **P121** | 1.04 | 0.94 | 0.46 | 0.09 | -0.79 | 0.18 | 0.49 | 0.44 (0.21-0.63) |
| **P122** | 1.51 | 1.07 | -0.05 | 0.09 | -1.24 | 0.18 | 0.20 | 0.64 (0.46-0.77) |
| **P123** | 0.96 | 0.98 | 0.59 | 0.09 | -0.82 | 0.18 | 0.28 | 0.68 (0.51-0.80) |
| **P124** | 0.80 | 0.93 | 0.94 | 0.09 | -0.13 | 0.18 | 0.59 | 0.55 (0.34-0.70) |
| **P125** | 0.31 | 0.61 | 2.13 | 0.09 | 4.31 | 0.18 | 0.45 | 0.46 (0.23-0.64) |
| **P126** | 0.66 | 0.85 | 1.08 | 0.09 | 0.25 | 0.18 | 0.46 | 0.48 (0.25-0.66) |
| **P127** | 0.78 | 0.99 | 1.00 | 0.09 | -0.25 | 0.18 | 0.41 | 0.59 (0.39-0.73) |
| **P128** | 0.62 | 0.83 | 1.19 | 0.09 | 0.58 | 0.18 | 0.55 | 0.65 (0.47-0.78) |
| **P129** | 0.46 | 0.79 | 1.67 | 0.09 | 1.89 | 0.18 | 0.37 | 0.68 (0.52-0.80) |
| **P130** | 1.89 | 1.00 | -0.47 | 0.09 | -0.88 | 0.18 | 0.37 | 0.55 (0.34-0.71) |
| **P131R** | 1.63 | 0.85 | 0.06 | 0.09 | -0.71 | 0.18 | 0.23 | 0.48 (0.25-0.65) |
| **P132** | 1.34 | 0.99 | 0.19 | 0.09 | -0.99 | 0.18 | 0.47 | 0.55 (0.35-0.71) |
| **P133** | 0.74 | 0.85 | 0.91 | 0.09 | -0.04 | 0.18 | 0.60 | 0.39 (0.15-0.59) |
| **P134** | 0.20 | 0.54 | 3.10 | 0.09 | 10.37 | 0.18 | 0.39 | 0.38 (0.13-0.58) |
| **P135** | 1.35 | 1.00 | 0.14 | 0.09 | -1.06 | 0.18 | 0.26 | 0.38 (0.14-0.57) |
| **P136** | 0.64 | 0.85 | 1.17 | 0.09 | 0.47 | 0.18 | 0.47 | 0.66 (0.48-0.78) |
| **P137** | 0.74 | 0.87 | 0.89 | 0.09 | -0.20 | 0.18 | 0.59 | 0.33 (0.09-0.54) |
| **P138** | 0.83 | 0.94 | 0.84 | 0.09 | -0.37 | 0.18 | 0.61 | 0.60 (0.41-0.74) |
| **P139** | 0.21 | 0.57 | 3.05 | 0.09 | 9.14 | 0.18 | 0.32 | 0.18 (-0.08-0.42) |
| **P140** | 1.27 | 0.95 | 0.18 | 0.09 | -0.94 | 0.18 | 0.35 | 0.34 (0.09-0.55) |
| **P141** | 0.74 | 0.94 | 1.00 | 0.09 | -0.13 | 0.18 | 0.59 | 0.63 (0.45-0.76) |
| **P142R** | 0.70 | 0.84 | 1.04 | 0.09 | 0.36 | 0.18 | 0.11 | 0.28 (0.03-0.50) |
| **P143** | 0.51 | 0.89 | 1.63 | 0.09 | 1.50 | 0.18 | 0.12 | 0.43 (0.19-0.61) |
| **P144** | 1.00 | 0.96 | 0.56 | 0.09 | -0.76 | 0.18 | 0.50 | 0.56 (0.35-0.72) |
| **P145** | 0.59 | 0.94 | 1.38 | 0.09 | 0.62 | 0.18 | 0.26 | 0.46 (0.23-0.64) |
| **P146** | 0.37 | 0.72 | 1.93 | 0.09 | 2.98 | 0.18 | 0.37 | 0.71 (0.56-0.82) |
| **P147** | 0.85 | 0.94 | 0.75 | 0.09 | -0.56 | 0.18 | 0.34 | 0.32 (0.11-0.55) |
| **P148** | 0.32 | 0.74 | 2.38 | 0.09 | 4.75 | 0.18 | 0.47 | 0.28 (0.03-0.50) |
| **P149** | 0.57 | 0.83 | 1.39 | 0.09 | 1.11 | 0.18 | 0.40 | 0.37 (0.12-0.57) |
| **P150** | 0.70 | 0.95 | 1.04 | 0.09 | -0.21 | 0.18 | 0.31 | 0.35 (0.10-0.55) |
| **P151** | 0.48 | 0.82 | 1.62 | 0.09 | 1.65 | 0.18 | 0.63 | 0.65 (0.47-0.77) |
| **P152** | 0.48 | 0.79 | 1.57 | 0.09 | 1.58 | 0.18 | 0.62 | 0.62 (0.43-0.76) |
| **P153** | 0.24 | 0.60 | 2.84 | 0.09 | 8.19 | 0.18 | 0.40 | 0.87 (0.80-0.92) |
| **P154** | 0.35 | 0.67 | 1.97 | 0.09 | 3.18 | 0.18 | 0.44 | 0.49 (0.27-0.66) |
| **P155R** | 1.26 | 0.98 | 0.36 | 0.09 | -0.84 | 0.18 | 0.40 | 0.36 (0.11-0.56) |
| **P156** | 0.41 | 0.69 | 1.69 | 0.09 | 2.43 | 0.18 | 0.43 | 0.46 (0.23-0.64) |
| **P157** | 0.51 | 0.83 | 1.56 | 0.09 | 1.53 | 0.18 | 0.59 | 0.68 (0.52-0.80) |
| **P158** | 0.96 | 0.96 | 0.68 | 0.09 | -0.58 | 0.18 | 0.49 | 0.55 (0.35-0.71) |
| **P159** | 0.51 | 0.74 | 1.42 | 0.09 | 1.49 | 0.18 | 0.40 | 0.40 (0.17-0.60) |
| **P160** | 0.35 | 0.70 | 2.08 | 0.09 | 3.80 | 0.18 | 0.29 | 0.48 (0.27-0.66) |
| **P161** | 0.58 | 0.76 | 1.09 | 0.09 | 0.28 | 0.18 | 0.51 | 0.69 (0.53-0.81) |
| **P162** | 0.36 | 0.73 | 2.08 | 0.09 | 3.60 | 0.18 | 0.47 | 0.57 (0.37-0.72) |
| **P163** | 0.46 | 0.79 | 1.66 | 0.09 | 1.81 | 0.18 | 0.60 | 0.75 (0.40-0.74) |
| **P164R** | 1.99 | 0.94 | -0.60 | 0.09 | -0.57 | 0.18 | -0.20 | 0.10 (-0.16-0.34) |
| **P165** | 1.30 | 1.07 | 0.25 | 0.09 | -1.19 | 0.18 | 0.17 | 0.38 (0.13-0.57) |
| **P166** | 0.25 | 0.62 | 2.65 | 0.09 | 6.63 | 0.18 | 0.41 | 0.76 (0.63-0.85) |
| **P167** | 0.94 | 0.90 | 0.59 | 0.09 | -0.62 | 0.18 | 0.35 | 0.13 (-0.13-0.37) |
| **P168** | 0.69 | 0.92 | 1.12 | 0.09 | 0.16 | 0.18 | 0.59 | 0.59 (0.40-0.74) |
| **P169** | 0.26 | 0.64 | 2.68 | 0.09 | 6.85 | 0.18 | 0.49 | 0.49 (0.27-0.66) |
| **P170** | 0.42 | 0.73 | 1.82 | 0.09 | 2.77 | 0.18 | 0.41 | 0.56 (0.35-0.72) |
| **P171** | 0.74 | 0.90 | 0.95 | 0.09 | -0.15 | 0.18 | 0.51 | 0.65 (0.48-0.78) |
| **P172** | 0.46 | 0.79 | 1.67 | 0.09 | 1.89 | 0.18 | 0.60 | 0.68 (0.51-0.79) |
| **P173** | 0.56 | 0.80 | 1.28 | 0.09 | 0.76 | 0.18 | 0.41 | 0.38 (0.14-0.58) |
| **P174** | 1.15 | 1.06 | 0.43 | 0.09 | -1.08 | 0.18 | 0.56 | 0.65 (0.47-0.77) |
| **P175** | 0.85 | 0.96 | 0.83 | 0.09 | -0.41 | 0.18 | 0.33 | 0.22 (-0.04-0.45) |
| **P176** | 1.26 | 1.04 | 0.22 | 0.09 | -1.17 | 0.18 | 0.30 | 0.39 (0.15-0.59) |
| **P177R** | 1.72 | 1.04 | -0.22 | 0.09 | -1.14 | 0.18 | -0.18 | 0.29 (0.03-0.51) |
| **P178** | 0.17 | 0.54 | 3.68 | 0.09 | 13.80 | 0.18 | 0.41 | 0.37 (0.13-0.57) |
| **P179** | 1.11 | 0.97 | 0.41 | 0.09 | -0.88 | 0.18 | 0.20 | 0.30 (0.05-0.51) |
| **P180** | 0.62 | 0.83 | 1.13 | 0.09 | 0.30 | 0.18 | 0.38 | 0.44 (0.21-0.62) |
| **P181** | 0.93 | 0.94 | 0.62 | 0.09 | -0.68 | 0.18 | 0.57 | 0.50 (0.25-0.64) |
| **P182** | 0.48 | 0.80 | 1.53 | 0.09 | 1.35 | 0.18 | 0.45 | 0.71 (0.56-0.82) |
| **P183** | 0.31 | 0.65 | 2.15 | 0.09 | 4.11 | 0.18 | 0.39 | 0.36 (0.12-0.56) |
| **P184** | 0.67 | 0.84 | 1.01 | 0.09 | 0.04 | 0.18 | 0.36 | 0.63 (0.45-0.77) |
| **P185** | 0.47 | 0.79 | 1.64 | 0.09 | 1.86 | 0.18 | 0.54 | 0.69 (0.53-0.80) |
| **P186** | 0.78 | 0.97 | 0.95 | 0.09 | -0.30 | 0.18 | 0.40 | 0.58 (0.38-0.72) |
| **P187** | 0.48 | 0.85 | 1.65 | 0.09 | 1.59 | 0.18 | 0.28 | 0.71 (0.56-0.82) |
| **P188** | 1.27 | 0.96 | 0.26 | 0.09 | -0.89 | 0.18 | 0.44 | 0.53 (0.32-0.69) |
| **P189** | 0.77 | 0.91 | 0.92 | 0.09 | -0.18 | 0.18 | 0.49 | 0.56 (0.36-0.71) |
| **P190** | 0.78 | 0.90 | 0.84 | 0.09 | -0.35 | 0.18 | 0.51 | 0.46 (0.24-0.64) |
| **P191** | 0.76 | 0.90 | 0.91 | 0.09 | -0.20 | 0.18 | 0.45 | 0.38 (0.14-0.58) |
| **P192** | 0.25 | 0.59 | 2.35 | 0.09 | 4.81 | 0.18 | 0.41 | 0.48 (0.26-0.65) |
| **P193** | 0.89 | 1.01 | 0.68 | 0.09 | -0.85 | 0.18 | 0.60 | 0.75 (0.61-0.84) |
| **P194** | 0.61 | 0.90 | 1.30 | 0.09 | 0.58 | 0.18 | 0.54 | 0.64 (0.46-0.77) |
| **P195** | 0.50 | 0.80 | 1.54 | 0.09 | 1.60 | 0.18 | 0.32 | 0.49 (0.27-0.66) |
| **P196** | 0.92 | 0.93 | 0.65 | 0.09 | -0.62 | 0.18 | 0.28 | 0.48 (0.26-0.65) |
| **P197** | 0.34 | 0.68 | 2.12 | 0.09 | 3.99 | 0.18 | 0.37 | 0.41 (0.18-0.60) |
| **P198** | 0.06 | 0.29 | 5.36 | 0.09 | 29.51 | 0.18 | 0.24 | 0.02 (-0.24-0.27) |
| **P199** | 0.84 | 0.94 | 0.84 | 0.09 | -0.32 | 0.18 | 0.50 | 0.30 (0.05-0.51) |
| **P200** | 0.24 | 0.57 | 2.71 | 0.09 | 7.62 | 0.18 | 0.48 | 0.34 (0.10-0.55) |
| **P201** | 0.53 | 0.83 | 1.44 | 0.09 | 1.11 | 0.18 | 0.36 | 0.42 (0.18-0.60) |
| **P202** | 0.79 | 0.88 | 0.76 | 0.09 | -0.49 | 0.18 | 0.35 | 0.53 (0.32-0.69) |
| **P203** | 0.47 | 0.82 | 1.70 | 0.09 | 1.92 | 0.18 | 0.29 | 0.39 (0.15-0.59) |
| **P204** | 0.92 | 0.95 | 0.69 | 0.09 | -0.57 | 0.18 | 0.44 | 0.42 (0.19-0.61) |
| **P205** | 0.61 | 0.88 | 1.28 | 0.09 | 0.60 | 0.18 | 0.59 | 0.60 (0.41-0.74) |
| **P206** | 0.25 | 0.56 | 2.36 | 0.09 | 5.55 | 0.18 | 0.44 | 0.69 (0.53-0.81) |
| **P207** | 0.31 | 0.65 | 2.29 | 0.09 | 5.00 | 0.18 | 0.37 | 0.42 (0.18-0.61) |
| **P208** | 0.31 | 0.65 | 2.22 | 0.09 | 4.63 | 0.18 | 0.45 | 0.34 (0.10-0.55) |
| **P209** | 0.68 | 0.98 | 1.17 | 0.09 | 0.04 | 0.18 | 0.49 | 0.68 (0.52-0.80) |
| **P210R** | 0.34 | 0.62 | 1.94 | 0.09 | 3.75 | 0.18 | 0.27 | 0.37 (0.14-0.58) |
| **P211** | 0.62 | 0.88 | 1.25 | 0.09 | 0.52 | 0.18 | 0.41 | 0.44 (0.21-0.62) |
| **P212** | 0.71 | 0.92 | 1.08 | 0.09 | 0.08 | 0.18 | 0.57 | 0.63 (0.44-0.76) |
| **P213** | 0.57 | 0.84 | 1.31 | 0.09 | 0.75 | 0.18 | 0.55 | 0.38 (0.14-0.58) |
| **P214** | 0.55 | 0.88 | 1.50 | 0.09 | 1.24 | 0.18 | 0.41 | 0.66 (0.49-0.78) |
| **P215R** | 1.42 | 1.05 | 0.07 | 0.09 | -1.20 | 0.18 | -0.06 | 0.20 (-0.66-0.43) |
| **P216** | 0.62 | 0.89 | 1.28 | 0.09 | 0.60 | 0.18 | 0.42 | 0.56 (0.36-0.71) |
| **P217** | 0.29 | 0.63 | 2.33 | 0.09 | 5.13 | 0.18 | 0.47 | 0.19 (-0.07-0.43) |
| **P218** | 0.36 | 0.67 | 1.86 | 0.09 | 2.86 | 0.18 | 0.46 | 0.69 (0.53-0.80) |
| **P219** | 0.21 | 0.52 | 2.80 | 0.09 | 8.36 | 0.18 | 0.43 | 0.78 (0.66-0.86) |
| **P220** | 1.19 | 1.00 | 0.25 | 0.09 | -1.11 | 0.18 | 0.22 | 0.64 (0.46-0.77) |

Ass = Asymmetry; Kurt = kurtosis; SD = Standard Deviation; SE= Standard Error; ICC = Intraclass Correlation Coefficient; R= Reversed scores, already computed; T/R = Test-Retest Reliability.

Supplementary Material SM3: Percentage of responses in the PID-5 (paper-and-pencil) different categories (N=730)

| **Item** | **Scores** | | | |
| --- | --- | --- | --- | --- |
|  | **0** | **1** | **2** | **3** |
| **P1** | 30.3 | 26.2 | 34.4 | 9.2 |
| **P2** | 75.1 | 13.3 | 8.8 | 2.9 |
| **P3** | 74.5 | 17.8 | 5.9 | 1.8 |
| **P4** | 37.7 | 33.8 | 21.9 | 6.6 |
| **P5** | 44.9 | 24.2 | 19.6 | 11.2 |
| **P6** | 31.4 | 33.2 | 23.6 | 11.9 |
| **P7R** | 41.1 | 32.3 | 19.5 | 7.1 |
| **P8** | 43.7 | 24.1 | 21.5 | 10.7 |
| **P9** | 30.7 | 33.8 | 27.4 | 8.1 |
| **P10** | 45.1 | 28.6 | 19.6 | 6.7 |
| **P11** | 92.9 | 3.6 | 1.1 | 2.5 |
| **P12** | 43.7 | 25.8 | 16.2 | 14.4 |
| **P13** | 68.2 | 21.2 | 9.0 | 1.5 |
| **P14** | 59.2 | 25.8 | 12.6 | 2.5 |
| **P15** | 47.3 | 30.8 | 18.1 | 3.8 |
| **P16** | 48.2 | 32.5 | 15.8 | 3.6 |
| **P17** | 52.7 | 31.0 | 12.6 | 3.7 |
| **P18** | 28.6 | 29.3 | 26.8 | 15.2 |
| **P19** | 80.1 | 12.5 | 6.2 | 1.2 |
| **P20** | 28.8 | 25.9 | 26.7 | 18.6 |
| **P21** | 51.2 | 27.0 | 15.5 | 6.3 |
| **P22** | 39.9 | 38.2 | 18.5 | 3.4 |
| **P23** | 54.4 | 26.4 | 12.9 | 6.3 |
| **P24** | 58.6 | 25.5 | 11.6 | 4.2 |
| **P25** | 64.0 | 21.0 | 10.7 | 4.4 |
| **P26** | 51.5 | 25.6 | 14.7 | 8.2 |
| **P27** | 54.0 | 22.7 | 14.9 | 8.4 |
| **P28** | 37.7 | 36.3 | 17.4 | 8.6 |
| **P29** | 51.0 | 32.2 | 13.6 | 3.3 |
| **P30R** | 25.9 | 39.5 | 22.7 | 11.9 |
| **P31** | 79.3 | 15.5 | 3.8 | 1.4 |
| **P32** | 40.8 | 27.1 | 21.0 | 11.1 |
| **P33** | 52.3 | 25.8 | 14.2 | 7.7 |
| **P34** | 39.3 | 25.6 | 25.1 | 10.0 |
| **P35R** | 19.6 | 19.3 | 27.8 | 33.3 |
| **P36** | 67.0 | 19.5 | 8.8 | 4.8 |
| **P37** | 87.5 | 7.0 | 3.7 | 1.8 |
| **P38** | 25.9 | 34.9 | 26.0 | 13.2 |
| **P39** | 75.2 | 16.6 | 5.5 | 2.7 |
| **P40** | 71.9 | 15.1 | 7.5 | 5.5 |
| **P41** | 79.3 | 14.1 | 4.8 | 1.8 |
| **P42** | 65.2 | 22.7 | 8.8 | 3.3 |
| **P43** | 56.7 | 26.6 | 13.2 | 3.6 |
| **P44** | 80.0 | 12.6 | 6.0 | 1.4 |
| **P45** | 41.8 | 33.7 | 18.2 | 6.3 |
| **P46** | 41.4 | 33.7 | 16.3 | 8.6 |
| **P47** | 45.6 | 28.5 | 18.2 | 7.7 |
| **P48** | 65.1 | 23.8 | 8.5 | 2.6 |
| **P49** | 25.3 | 26.4 | 31.2 | 17.0 |
| **P50** | 36.0 | 28.5 | 24.0 | 11.5 |
| **P51** | 31.6 | 30.0 | 24.8 | 13.6 |
| **P52** | 51.0 | 27.5 | 15.3 | 6.2 |
| **P53** | 82.2 | 12.5 | 4.1 | 1.2 |
| **P54** | 84.9 | 9.3 | 3.4 | 2.3 |
| **P55** | 68.5 | 20.4 | 8.4 | 2.7 |
| **P56** | 76.6 | 17.1 | 4.9 | 1.4 |
| **P57** | 83.3 | 8.9 | 4.8 | 3.0 |
| **P58R** | 45.8 | 32.6 | 15.5 | 6.2 |
| **P59** | 51.0 | 22.5 | 15.9 | 10.7 |
| **P60** | 47.9 | 35.9 | 13.6 | 2.6 |
| **P61** | 41.8 | 27.1 | 19.9 | 11.2 |
| **P62** | 41.8 | 27.8 | 20.0 | 10.4 |
| **P63** | 47.1 | 36.3 | 14.4 | 2.2 |
| **P64** | 71.5 | 17.4 | 8.1 | 3.0 |
| **P65** | 46.3 | 25.1 | 22.1 | 6.6 |
| **P66** | 70.4 | 16.2 | 7.9 | 5.5 |
| **P67** | 61.8 | 25.2 | 11.1 | 1.9 |
| **P68** | 52.1 | 29.0 | 15.8 | 3.2 |
| **P69** | 31.6 | 33.8 | 27.5 | 7.0 |
| **P70** | 62.9 | 22.3 | 11.6 | 3.2 |
| **P71** | 63.7 | 20.7 | 12.5 | 3.2 |
| **P72** | 83.0 | 10.1 | 5.2 | 1.6 |
| **P73** | 82.7 | 12.7 | 3.8 | 0.7 |
| **P74** | 62.7 | 22.7 | 12.6 | 1.9 |
| **P75** | 50.0 | 30.8 | 15.6 | 3.6 |
| **P76** | 64.5 | 21.4 | 11.2 | 2.9 |
| **P77** | 74.7 | 15.9 | 8.4 | 1.1 |
| **P78** | 51.1 | 30.1 | 15.8 | 3.0 |
| **P79** | 23.0 | 27.1 | 29.3 | 20.5 |
| **P80** | 43.7 | 30.8 | 20.5 | 4.9 |
| **P81** | 86.0 | 7.0 | 4.9 | 2.1 |
| **P82** | 55.9 | 25.8 | 15.3 | 3.0 |
| **P83** | 41.4 | 30.3 | 19.2 | 9.2 |
| **P84** | 73.4 | 13.3 | 10.3 | 3.0 |
| **P85** | 52.6 | 31.8 | 11.1 | 4.5 |
| **P86** | 44.9 | 26.0 | 18.1 | 11.0 |
| **P87R** | 16.7 | 21.0 | 31.9 | 30.4 |
| **P88** | 48.2 | 32.2 | 14.7 | 4.9 |
| **P89** | 69.2 | 16.8 | 7.9 | 6.0 |
| **P90R** | 52.3 | 11.9 | 18.2 | 17.5 |
| **P91** | 41.1 | 27.1 | 21.9 | 9.9 |
| **P92** | 33.4 | 28.8 | 25.8 | 12.1 |
| **P93** | 41.2 | 25.8 | 19.7 | 13.3 |
| **P94** | 51.5 | 22.2 | 18.4 | 7.9 |
| **P95** | 35.3 | 29.7 | 22.7 | 12.2 |
| **P96R** | 5.1 | 12.2 | 23.8 | 58.9 |
| **P97R** | 42.6 | 32.5 | 13.4 | 11.5 |
| **P98R** | 51.9 | 30.3 | 13.6 | 4.2 |
| **P99** | 60.5 | 13.6 | 16.3 | 9.6 |
| **P100** | 54.8 | 25.2 | 14.2 | 5.8 |
| **P101** | 40.0 | 27.4 | 21.5 | 11.1 |
| **P102** | 13.2 | 22.5 | 33.6 | 30.8 |
| **P103** | 27.9 | 26.2 | 28.1 | 17.8 |
| **P104** | 53.4 | 24.4 | 14.8 | 7.4 |
| **P105** | 41.5 | 31.8 | 21.5 | 5.2 |
| **P106** | 68.6 | 15.3 | 10.3 | 5.8 |
| **P107** | 46.6 | 28.8 | 20.5 | 4.1 |
| **P108** | 73.4 | 15.5 | 7.3 | 3.8 |
| **P109** | 11.0 | 24.9 | 34.4 | 29.7 |
| **P110** | 16.4 | 22.1 | 33.6 | 27.9 |
| **P111** | 48.8 | 23.7 | 19.9 | 7.7 |
| **P112** | 22.5 | 31.2 | 36.7 | 9.6 |
| **P113** | 59.6 | 26.3 | 11.8 | 2.3 |
| **P114** | 71.1 | 17.4 | 8.9 | 2.6 |
| **P115** | 45.3 | 27.1 | 17.9 | 9.6 |
| **P116** | 66.3 | 23.2 | 8.2 | 2.3 |
| **P117** | 17.3 | 27.4 | 30.7 | 24.7 |
| **P118** | 36.0 | 29.9 | 24.0 | 10.1 |
| **P119** | 83.4 | 9.2 | 4.4 | 3.0 |
| **P120** | 56.3 | 21.9 | 14.1 | 7.7 |
| **P121** | 34.4 | 34.4 | 23.8 | 7.4 |
| **P122** | 22.9 | 25.2 | 30.3 | 21.6 |
| **P123** | 41.9 | 28.1 | 22.1 | 7.9 |
| **P124** | 48.8 | 30.0 | 14.1 | 7.1 |
| **P125** | 76.6 | 17.4 | 4.9 | 1.1 |
| **P126** | 54.7 | 28.2 | 13.3 | 3.8 |
| **P127** | 54.0 | 23.0 | 14.2 | 8.8 |
| **P128** | 56.4 | 28.4 | 11.5 | 3.7 |
| **P129** | 69.3 | 18.4 | 9.0 | 3.3 |
| **P130** | 11.2 | 21.8 | 33.4 | 33.6 |
| **P131R** | 7.4 | 39.0 | 36.4 | 17.1 |
| **P132** | 22.7 | 34.8 | 27.8 | 14.7 |
| **P133** | 48.8 | 32.6 | 14.8 | 3.8 |
| **P134** | 84.7 | 11.6 | 2.3 | 1.4 |
| **P135** | 23.8 | 32.1 | 29.2 | 14.9 |
| **P136** | 56.2 | 27.7 | 12.1 | 4.1 |
| **P137** | 50.1 | 29.5 | 16.3 | 4.1 |
| **P138** | 47.5 | 29.0 | 16.7 | 6.7 |
| **P139** | 86.3 | 8.2 | 4.1 | 1.4 |
| **P140** | 24.5 | 34.2 | 30.5 | 10.7 |
| **P141** | 53.6 | 24.9 | 15.1 | 6.4 |
| **P142R** | 50.4 | 33.6 | 11.9 | 4.1 |
| **P143** | 69.6 | 15.5 | 8.9 | 6.0 |
| **P144** | 37.8 | 32.5 | 21.5 | 8.2 |
| **P145** | 65.8 | 15.8 | 11.9 | 6.6 |
| **P146** | 74.4 | 15.9 | 7.7 | 2.1 |
| **P147** | 46.7 | 27.9 | 19.5 | 5.9 |
| **P148** | 81.2 | 9.2 | 6.2 | 3.4 |
| **P149** | 61.2 | 24.9 | 9.6 | 4.2 |
| **P150** | 58.9 | 18.2 | 17.1 | 5.8 |
| **P151** | 68.8 | 17.8 | 9.6 | 3.8 |
| **P152** | 67.7 | 19.6 | 9.7 | 3.0 |
| **P153** | 83.0 | 11.9 | 3.3 | 1.8 |
| **P154** | 75.5 | 15.9 | 7.3 | 1.4 |
| **P155R** | 24.2 | 39.6 | 22.5 | 13.7 |
| **P156** | 68.8 | 23.0 | 6.6 | 1.6 |
| **P157** | 66.3 | 20.4 | 9.2 | 4.1 |
| **P158** | 39.6 | 33.7 | 17.8 | 8.9 |
| **P159** | 61.6 | 28.1 | 7.9 | 2.3 |
| **P160** | 75.5 | 16.2 | 6.2 | 2.2 |
| **P161** | 57.5 | 28.8 | 12.2 | 1.5 |
| **P162** | 76.4 | 14.0 | 7.0 | 2.6 |
| **P163** | 69.9 | 17.4 | 9.6 | 3.2 |
| **P164R** | 8.5 | 19.0 | 37.3 | 35.2 |
| **P165** | 28.6 | 30.0 | 23.7 | 17.7 |
| **P166** | 83.2 | 10.1 | 5.1 | 1.6 |
| **P167** | 38.6 | 34.8 | 21.0 | 5.6 |
| **P168** | 56.3 | 24.4 | 13.4 | 5.9 |
| **P169** | 82.7 | 10.7 | 4.5 | 2.1 |
| **P170** | 70.1 | 20.7 | 6.6 | 2.6 |
| **P171** | 52.2 | 27.0 | 15.9 | 4.9 |
| **P172** | 69.6 | 18.1 | 9.2 | 3.2 |
| **P173** | 61.1 | 24.7 | 11.5 | 2.7 |
| **P174** | 35.5 | 28.6 | 21.4 | 14.5 |
| **P175** | 46.6 | 29.3 | 16.3 | 7.8 |
| **P176** | 30.5 | 27.3 | 28.1 | 14.1 |
| **P177R** | 14.8 | 27.3 | 29.2 | 28.8 |
| **P178** | 89.3 | 6.4 | 2.5 | 1.8 |
| **P179** | 32.6 | 33.6 | 24.5 | 9.3 |
| **P180** | 57.7 | 25.6 | 13.6 | 3.2 |
| **P181** | 41.1 | 31.2 | 20.8 | 6.8 |
| **P182** | 68.4 | 17.9 | 11.0 | 2.7 |
| **P183** | 77.5 | 14.9 | 6.2 | 1.4 |
| **P184** | 54.0 | 28.2 | 14.7 | 3.2 |
| **P185** | 68.5 | 19.5 | 8.9 | 3.2 |
| **P186** | 52.7 | 24.0 | 15.6 | 7.7 |
| **P187** | 71.0 | 14.4 | 10.4 | 4.2 |
| **P188** | 24.0 | 36.8 | 27.1 | 12.1 |
| **P189** | 50.0 | 28.8 | 15.8 | 5.5 |
| **P190** | 48.8 | 28.9 | 17.5 | 4.8 |
| **P191** | 50.4 | 28.5 | 16.2 | 4.9 |
| **P192** | 81.8 | 11.6 | 6.0 | 0.5 |
| **P193** | 48.6 | 21.4 | 22.2 | 7.8 |
| **P194** | 61.9 | 20.5 | 12.1 | 5.5 |
| **P195** | 65.2 | 22.6 | 8.8 | 3.4 |
| **P196** | 41.2 | 32.1 | 20.1 | 6.6 |
| **P197** | 76.2 | 15.9 | 6.0 | 1.9 |
| **P198** | 95.6 | 2.9 | 1.5 | 0.0 |
| **P199** | 46.3 | 30.7 | 16.0 | 7.0 |
| **P200** | 82.3 | 13.0 | 3.4 | 1.2 |
| **P201** | 65.2 | 20.3 | 11.0 | 3.6 |
| **P202** | 47.7 | 29.5 | 19.0 | 3.8 |
| **P203** | 69.9 | 17.3 | 8.8 | 4.1 |
| **P204** | 41.8 | 31.9 | 18.8 | 7.5 |
| **P205** | 60.8 | 22.2 | 11.9 | 5.1 |
| **P206** | 80.1 | 15.2 | 4.0 | 0.7 |
| **P207** | 77.9 | 15.3 | 4.9 | 1.8 |
| **P208** | 77.3 | 15.9 | 5.2 | 1.6 |
| **P209** | 61.4 | 17.0 | 14.0 | 7.7 |
| **P210R** | 72.6 | 21.9 | 4.2 | 1.2 |
| **P211** | 60.3 | 22.6 | 12.5 | 4.7 |
| **P212** | 55.2 | 24.9 | 13.8 | 6.0 |
| **P213** | 61.8 | 22.7 | 11.8 | 3.7 |
| **P214** | 64.8 | 20.5 | 9.0 | 5.6 |
| **P215R** | 24.4 | 27.9 | 29.0 | 18.6 |
| **P216** | 60.1 | 22.9 | 11.4 | 5.6 |
| **P217** | 78.9 | 14.5 | 5.1 | 1.5 |
| **P218** | 73.3 | 18.6 | 6.8 | 1.2 |
| **P219** | 83.4 | 12.9 | 2.9 | 0.8 |
| **P220** | 31.9 | 27.8 | 29.6 | 10.7 |

0 = Very false or often false; 1 = Sometimes or somewhat false; 2 = Sometimes or somewhat true; 3 = Very true or often true; R = Reverse scores already computed.

Supplementary Material SM4: Correlations between PID-5 (paper-and-pencil) facets (N=730)

|  | **1** | **2** | **3** | **4** | **5** | **6** | **7** | **8** | **9** | **10** | **11** | **12** | **13** | **14** | **15** | **16** | **17** | **18** | **19** | **20** | **21** | **22** | **23** | **24** | **25** |
| --- | --- | --- | --- | --- | --- | --- | --- | --- | --- | --- | --- | --- | --- | --- | --- | --- | --- | --- | --- | --- | --- | --- | --- | --- | --- |
| **1** | **-** |  |  |  |  |  |  |  |  |  |  |  |  |  |  |  |  |  |  |  |  |  |  |  |  |
| **2** | **0.57^*^** | **-** |  |  |  |  |  |  |  |  |  |  |  |  |  |  |  |  |  |  |  |  |  |  |  |
| **3** | 0.48^*^ | **0.55^*^** | **-** |  |  |  |  |  |  |  |  |  |  |  |  |  |  |  |  |  |  |  |  |  |  |
| **4** | 0.28^*^ | 0.42^*^ | 0.32^*^ | **-** |  |  |  |  |  |  |  |  |  |  |  |  |  |  |  |  |  |  |  | - |  |
| **5** | 0.42^*^ | 0.45^*^ | 0.37^*^ | 0.21^*^ | - |  |  |  |  |  |  |  |  |  |  |  |  |  |  |  |  |  |  |  |  |
| **6** | 0.47^*^ | **0.59^*^** | 0.48^*^ | 0.42^*^ | 0.48^*^ | **-** |  |  |  |  |  |  |  |  |  |  |  |  |  |  |  |  |  |  |  |
| **7** | 0.19^*^ | 0.38^*^ | 0.20^*^ | 0.27^*^ | 0.46^*^ | 0.43^*^ | **-** |  |  |  |  |  |  |  |  |  |  |  |  |  |  |  |  |  |  |
| **8** | 0.01 | 0.01^*^ | -0.03 | 0.04 | 0.19^*^ | **0.62^*^** | 0.30^*^ | - |  |  |  |  |  |  |  |  |  |  |  |  |  |  |  |  |  |
| **9** | 0.40^*^ | **0.58^*^** | **0.51^*^** | 0.38^*^ | **0.45^*^** | **0.56^*^** | 0.32^*^ | 0.30^*^ | **-** |  |  |  |  |  |  |  |  |  |  |  |  |  |  |  |  |
| **10** | 0.44^*^ | **0.59^*^** | 0.45^*^ | 0.44^*^ | 0.42^*^ | **0.58^*^** | 0.39^*^ | 0.32^*^ | **0.76^*^** | **-** |  |  |  |  |  |  |  |  |  |  |  |  |  |  |  |
| **11** | -0.03 | 0.21^*^ | 0.12^*^ | 0.19^*^ | 0.37^*^ | 0.41^*^ | 0.14^*^ | 0.39^*^ | 0.40^*^ | 0.37^*^ | - |  |  |  |  |  |  |  |  |  |  |  |  |  |  |
| **12** | 0.36^*^ | 0.46^*^ | 0.38^*^ | 0.26^*^ | 0.46^*^ | 0.23^*^ | 0.01^*^ | 0.14^*^ | 0.46^*^ | 0.43^*^ | 0.32^*^ | **-** |  |  |  |  |  |  |  |  |  |  |  |  |  |
| **13** | 0.14^*^ | 0.22^*^ | 0.27^*^ | 0.28^*^ | 0.42^*^ | 0.32^*^ | 0.15^*^ | 0.10^*^ | 0.24^*^ | 0.28^*^ | 0.31^*^ | 0.32^*^ | **-** |  |  |  |  |  |  |  |  |  |  |  |  |
| **14** | 0.12^*^ | 0.23^*^ | 0.27^*^ | 0.31^*^ | 0.44^*^ | 0.30^*^ | 0.13^*^ | 0.15^*^ | 0.32^*^ | 0.39^*^ | 0.36^*^ | 0.37^*^ | **0.77^*^** | **-** |  |  |  |  |  |  |  |  |  |  |  |
| **15** | 0.15^*^ | 0.18^*^ | 0.23^*^ | 0.18^*^ | 0.40^*^ | 0.15^*^ | 0.04 | 0.13^*^ | 0.16^*^ | 0.13^*^ | 0.30^*^ | 0.26^*^ | **0.52^*^** | 0.43^*^ | - |  |  |  |  |  |  |  |  |  |  |
| **16** | 0.33^*^ | 0.34^*^ | 0.37^*^ | 0.37^*^ | 0.41^*^ | **0.50^*^** | 0.34^*^ | 0.04 | 0.24^*^ | 0.34^*^ | 0.17^*^ | 0.31^*^ | **0.59^*^** | **0.52^*^** | 0.48^*^ | - |  |  |  |  |  |  |  |  |  |
| **17** | 0.07^*^ | 0.17^*^ | 0.21^*^ | 0.17^*^ | **0.56^*^** | 0.37^*^ | 0.20^*^ | 0.34^*^ | 0.38^*^ | 0.37^*^ | **0.52^*^** | 0.37^*^ | **0.52^*^** | **0.60^*^** | 0.46^*^ | 0.33^*^ | **-** |  |  |  |  |  |  |  |  |
| **18** | 0.25^*^ | 0.25^*^ | 0.27^*^ | 0.37^*^ | 0.40^*^ | 0.17^*^ | 0.07 | 0.20^*^ | 0.38^*^ | 0.44^*^ | 0.36^*^ | 0.32^*^ | **0.49^*^** | **0.60^*^** | 0.28^*^ | 0.40^*^ | **0.52^*^** | **-** |  |  |  |  |  |  |  |
| **19** | 0.45^*^ | 0.35^*^ | 0.33^*^ | 0.24^*^ | 0.49^*^ | 0.39^*^ | 0.14^*^ | 0.07 | 0.29^*^ | 0.36^*^ | 0.13^*^ | 0.31^*^ | 0.26^*^ | 0.30^*^ | 0.14^*^ | 0.33^*^ | 0.31^*^ | 0.44^*^ | **-** |  |  |  |  |  |  |
| **20** | 0.44^*^ | 0.49^*^ | 0.38^*^ | 0.40^*^ | 0.39^*^ | 0.04 | 0.05 | 0.14^*^ | **0.54^*^** | **0.54^*^** | 0.28^*^ | 0.34^*^ | 0.31^*^ | 0.37^*^ | 0.20^*^ | 0.38^*^ | 0.32^*^ | **0.54^*^** | 0.46^*^ | **-** |  |  |  |  |  |
| **21** | -0.00 | -0.10^*^ | -0.05 | -0.03 | 0.16^*^ | 0.32^*^ | 0.12^*^ | 0.05 | -0.06 | 0.09^*^ | 0.22^*^ | 0.06 | 0.33^*^ | 0.31^*^ | 0.18^*^ | 0.24^*^ | **0.30^*^** | 0.31^*^ | 0.22^*^ | 0.09^*^ | **-** |  |  |  |  |
| **22** | 0.28^*^ | 0.42^*^ | 0.28^*^ | 0.18^*^ | 0.29^*^ | 0.31^*^ | 0.17^*^ | 0.12^*^ | 0.23^*^ | 0.19^*^ | 0.23^*^ | 0.33^*^ | 0.14^*^ | 0.08^*^ | 0.32^*^ | 0.20^*^ | 0.11^*^ | -0.01 | 0.06 | 0.12^*^ | -0.06 | - |  |  |  |
| **23** | 0.36^*^ | 0.34^*^ | 0.26^*^ | 0.18^*^ | 0.33^*^ | **0.52^*^** | 0.30^*^ | 0.17^*^ | 0.29^*^ | 0.33^*^ | 0.29^*^ | 0.35^*^ | 0.40^*^ | 0.31^*^ | 0.41^*^ | 0.34^*^ | 0.31^*^ | 0.33^*^ | 0.26^*^ | 0.33^*^ | 0.23^*^ | 0.32^*^ | - |  |  |
| **24** | 0.36^*^ | 0.39^*^ | 0.29^*^ | 0.27^*^ | 0.42^*^ | 0.44^*^ | 0.21^*^ | 0.30^*^ | 0.48^*^ | **0.62^*^** | 0.45^*^ | 0.39^*^ | 0.39^*^ | 0.45^*^ | 0.36^*^ | 0.38^*^ | **0.51^*^** | 0.47^*^ | 0.33^*^ | 0.44^*^ | 0.28^*^ | 0.27^*^ | **0.56^*^** | **-** |  |
| **25** | **0.51^*^** | **0.53^*^** | 0.47^*^ | 0.37^*^ | 0.46^*^ | 0.43^*^ | 0.30^*^ | 0.21^*^ | **0.54^*^** | **0.61^*^** | 0.37^*^ | 0.44^*^ | 0.42^*^ | 0.46^*^ | 0.37^*^ | 0.42^*^ | 0.42^*^ | **0.52^*^** | 0.39**^*^** | **0.56^*^** | 0.20^*^ | 0.34^*^ | **0.64^*^** | **0.68^*^** | **-** |

1 = Emotional Lability (NA); 2 = Anxiousness (NA), 3 = Separation Insecurity (AN), 4 = Submissiveness (AN), 5 = Hostility (AN), 6 = Perseveration (AN), 7 = Withdrawal (DIST), 8 = Intimacy Avoidance (DIST), 9 = Anhedonia (DIST), 10 = Depressivity (DIST), 11 = Restricted Affectivity (DIST), 12 = Suspiciousness (DIST), 13 = Manipulativeness (ANT), 14 = Deceitfulness (ANT), 15 = Grandiosity (ANT), 16 = Attention Seeking (ANT), 17 = Callousness (ANT), 18 = Irresponsibility (DES), 19 = Impulsivity (DES), 20 = Distractibility (DES), 21 = Risk Taking (DES), 22 = Rigid Perfectionism (DES), 23 = Unusual Beliefs & Experiences (PSI), 24 = Eccentricity (PSY), 25 = Perceptual Dysregulation (PSI); * =p≤0.05; Bold = ≥0.51 (Strong correlations according to the parameters established by Streiner and Norman, 2003).

Supplementary Material SM5: Indicators concerning the two-dimension analysis of PID-5 facets 5 (paper-and-pencil), the unidemensionality of which was rejected (N=730)

| **Two-dimensionality** | | | | | | |
| --- | --- | --- | --- | --- | --- | --- |
| **Domain** | **Facet** | **Factors** | **χ^2^ (df)** | **TLI** | **RMSEA** | **RMSR** |
| **NA** | **Emotional Lability** | F1: P102, P122, P165 F2: P18, P62, P138, P181 | 71 (8) | 0.941 | 0.104 | 0.02 |
| **NA** | **Anxiousness** | F1: P79, P93, P95, P141, P174 F2: P96, P109, P110, P130 | 120 (19) | 0.944 | 0.084 | 0.03 |
| **NA** | **Hostility** | F1: P28, P38, P92, P158, P188 F2: P32, P85, P116, P170, P216 | 160 (26) | 0.930 | 0.085 | 0.04 |
| **NA** | **Perseveration** | F1: P60, P78, P80, P121, P128, P137 F2: P46, P51, P100 | 110 (19) | 0.931 | 0.081 | 0.03 |
| **DET** | **Restricted Affectivity** | F1: P8, P45, P84, P91, P167, P184 F2: P101 | 41,9 (8) | 0.946 | 0.076 | 0.03 |
| **DET** | **Depressivity** | F1: P27, P61, P86, P104, P148, P163, P168, P169, P212 F2: P66, P81, P119, P151, P178 | 563 (64) | 0.927 | 0.103 | 0.03 |
| **DET** | **Suspiciousness** | F1: P2, P103, P133, P190 F2: P117, P131, P177 | 35,4 (8) | 0.909 | 0.068 | 0.03 |
| **ANT** | **Deceitfulness** | F1: P2, P103, P133, P190 F2: P117, P131, P177 | 35,4 (8) | 0.909 | 0.068 | 0.03 |
| **ANT** | **Callousness** | F1: P19, P54, P72, P153, P166, P183, P207, P208 F2: P11, P13, P73, P90, P198, P200 | 704,9 (64) | 0.853 | 0.117 | 0.05 |
| **DIS** | **Attention Seeking** | F1: P74, P111, P113, P173, P211 F2: P14, P43, P191 | 79 (13) | 0.963 | 0.083 | 0.02 |
| **DIS** | **Risk Taking** | F1: P3, P39, P48, P67, P69, P112, P159, P195 F2: P7, P35, P87, P98, P164, P215 | 314 (64) | 0.901 | 0.073 | 0.04 |
| **PSY** | **Unusual Beliefs** | F1: P99, P106, P139, P143, P209 F2: P94, P150, P194 | 87.8 (13) | 0.922 | 0.089 | 0.04 |
| **PSY** | **Perceptual Dysregulation** | F1: P37, P83, P154, P193, P213 F2: P36, P42, P44, P59, P77, P192, P217 | 388.8 (43) | 0.852 | 0.105 | 0.05 |

ANT = Antagonism; DET = Detachment; df: Degrees of freedom; DIS= Disinhibition; MI: Modification Index; NA = Negative Affect; PSY = Psychoticism; RMSEA: Root mean square error of approximation; RMSR = Root Mean Square Residual; TLI: Tucker-Lewis Index; X²= Chi-Square.

Supplementary Material SM6: Factor loadings of items regarding facets and facets regarding PID-5 (paper-and-pencil) domains according to Confirmatory Factor Analysis (N=730)

|  | **FACETS** | | | | | | | | | | |
| --- | --- | --- | --- | --- | --- | --- | --- | --- | --- | --- | --- |
| **Emotional Lability** | | | **Separation Insecurity** | | | **Suspiciousness** | | **Irresponsibility** | | **Restricted Affectivity** | |
| P18 | | **0.654** | P12 | **0.701** | | P2 | 0.232 | P31 | **0.486** | P8 | **0.639** |
| P62 | | **0.653** | P50 | **0.612** | | P103 | **0.594** | P129 | **0.574** | P45 | **0.507** |
| P102 | | **0.412** | P57 | 0.335 | | P117 | 0.274 | P156 | **0.569** | P84 | **0.495** |
| P122 | | **0.540** | P64 | **0.590** | | P131R | 0.264 | P160 | **0.372** | P91 | **0.608** |
| P138 | | **0.737** | P127 | **0.802** | | P133 | **0.722** | P171 | **0.653** | P101 | **0.600** |
| P165 | | **0.491** | P149 | **0.584** | | P177R | -0.129 | P201 | **0.456** | P167 | **0.554** |
| P181 | | **0.708** | P175 | **0.703** | | P190 | **0.626** | P210R | **0.383** | P184 | **0.612** |
| **Deceitfulness** | | | **Rigid Perfectionism** | | | **Anxiousness** | | **Perseveration** | | **Distractibility** | |
| P41 | | **0.436** | P34 | **0.481** | | P79 | **0.642** | P46 | **0.325** | P6 | **0.617** |
| P53 | | **0.580** | P49 | **0.634** | | P93 | **0.643** | P51 | **0.356** | P29 | **0.700** |
| P56 | | **0.594** | P105 | **0.566** | | P95 | **0.671** | P60 | **0.587** | P47 | **0.389** |
| P76 | | **0.707** | P115 | **0.606** | | P96R | 0.147 | P78 | **0.533** | P68 | **0.663** |
| P126 | | **0.492** | P123 | **0.780** | | P109 | **0.670** | P80 | **0.674** | P88 | **0.519** |
| P134 | | **0.573** | P135 | **0.644** | | P110 | **0.690** | P100 | **0.545** | P118 | **0.774** |
| P142R | | 0.198 | P140 | **0.525** | | P130 | **0.564** | P121 | **0.587** | P132 | **0.802** |
| P206 | | **0.735** | P176 | **0.706** | | P141 | **0.619** | P128 | **0.683** | P144 | **0.785** |
| P214 | | **0.684** | P196 | **0.618** | | P174 | **0.768** | P137 | **0.647** | P199 | **0.733** |
| P218 | | **0.686** | P220 | **0.565** | |  |  |  |  |  |  |
| **Hostility** | |  | **Withdrawal** | | | **Anhedonia** | | **Attention Seeking** | | **Unusual Beliefs & Experiences** | |
| P28 | | **0.731** | P10 | **0.711** | | P1 | **0.578** | P14 | **0.636** | P94 | **0.554** |
| P32 | | **0.477** | P20 | **0.597** | | P23 | **0.692** | P43 | **0.530** | P99 | **0.496** |
| P38 | | **0.798** | P75 | **0.667** | | P26 | **0.635** | P74 | **0.815** | P106 | **0.524** |
| P85 | | **0.438** | P82 | **0.806** | | P30R | 0.345 | P111 | **0.693** | P139 | **0.474** |
| P92 | | **0.726** | P136 | **0.607** | | P124 | **0.747** | P113 | **0.561** | P143 | 0.295 |
| P116 | | **0.463** | P146 | **0.577** | | P155R | **0.537** | P173 | **0.838** | P150 | **0.413** |
| P158 | | **0.790** | P147 | **0.570** | | P157 | **0.747** | P191 | **0.428** | P194 | **0.715** |
| P170 | | **0.352** | P161 | **0.779** | | P189 | **0.618** | P211 | **0.789** | P209 | **0.655** |
| P188 | | **0.616** | P182 | **0.609** | |  |  |  |  |  |  |
| P216 | | **0.489** | P186 | **0.658** | |  |  |  |  |  |  |
| **Intimacy Avoidance** | | | **Impulsivity** | | | **Grandiosity** | | **Manipulativeness** | | **Submissiveness** | |
| P89 | | **0.702** | P4 | **0.742** | | P40 | **0.632** | P107 | **0.617** | P9 | **0.628** |
| P97R | | **0.553** | P16 | **0.809** | | P65 | **0.502** | P125 | **0.630** | P15 | **0.751** |
| P108 | | **0.521** | P17 | **0.782** | | P114 | **0.775** | P162 | **0.722** | P63 | **0.688** |
| P120 | | **0.402** | P22 | **0.720** | | P179 | 0.348 | P180 | **0.564** | P202 | **0.667** |
| P145 | | **0.765** | P58R | **0.531** | | P187 | 0.389 | P219 | **0.747** |  |  |
| P203 | | **0.650** | P204 | **0.656** | | P197 | 0.563 |  |  |  |  |
| **Depressivity** | | | **Callousness** | | **Perceptual Dysregulation** | | | **Risk Taking** | | **Eccentricity** | |
| P27 | | **0.760** | P11 | 0.199 | | P36 | **0.466** | P3 | 0.366 | P5 | **0.701** |
| P61 | | **0.703** | P13 | **0.442** | | P37 | 0.344 | P7R | 0.371 | P21 | **0.741** |
| P66 | | **0.663** | P19 | **0.660** | | P42 | 0.357 | P35R | 0.324 | P24 | **0.749** |
| P81 | | **0.692** | P54 | **0.634** | | P44 | **0.445** | P39 | **0.550** | P25 | **0.792** |
| P86 | | **0.410** | P72 | **0.681** | | P59 | **0.519** | P48 | **0.590** | P33 | **0.732** |
| P104 | | **0.786** | P73 | **0.453** | | P77 | 0.398 | P67 | **0.694** | P52 | **0.696** |
| P119 | | **0.635** | P90R | 0.244 | | P83 | **0.568** | P69 | **0.538** | P55 | **0.694** |
| P148 | | **0.690** | P153 | **0.714** | | P154 | **0.505** | P87R | **0.410** | P70 | **0.769** |
| P151 | | **0.745** | P166 | **0.631** | | P192 | **0.474** | P98R | **0.415** | P71 | **0.734** |
| P163 | | **0.771** | P183 | **0.632** | | P193 | **0.684** | P112 | **0.612** | P152 | **0.820** |
| P168 | | **0.792** | P198 | 0.269 | | P213 | **0.643** | P159 | **0.651** | P172 | **0.796** |
| P169 | | **0.723** | P200 | **0.534** | | P217 | **0.561** | P164R | 0.292 | P185 | **0.708** |
| P178 | | **0.591** | P207 | **0.601** | |  |  | P195 | **0.407** | P205 | **0.798** |
|  | P212 | **0.661** | P208 | **0.551** | |  |  | P215R | **0.422** |  |  |

**DOMAINS**

| **Negative Affect** | | **Detachment** | | **Antagonism** | |
| --- | --- | --- | --- | --- | --- |
| Emotional Lability | **0.798** | Withdrawal | **0.742** | Manipulativeness | **1.005** |
| Anxiousness | **0.845** | Intimacy Avoidance | **0.401** | Deceitfulness | **0.988** |
| Separation Insecurity | **0.649** | Anhedonia | **0.935** | Grandiosity | **0.683** |
| Submissiveness | **0.570** | Depressivity | **0.878** | Attention Seeking | **0.609** |
| Hostility | **0.642** | Restricted Affectivity | **0.574** | Callousness | **0.658** |
| Perseveration | **0.873** | Suspiciousness | **0.813** |  |  |
|  |  |  |  |  |  |
| **Disinhibition** | | **Psychoticism** | |  |  |
| Irresponsibility | **0.842** | Unusual Beliefs & Experiences | **0.812** |  |  |
| Impulsivity | **0.608** | Eccentricity | **0.790** |  |  |
| Distractibility | **0.735** | Perceptual Dysregulation | **0.978** |  |  |
| Risk Taking | **0.399** |  |  |  |  |
| Rigid Perfectionism | **0.283** |  |  |  |  |

Supplementary Material SM7: Factor loadings of facets in the PID-5 (paper-and-pencil) domains according to Exploratory Factor Analysis – Four- and Six-factor Models (N=730)

|  | **4-factor Model** | | | |
| --- | --- | --- | --- | --- |
| **Facets** | **Factor 1** | **Factor 2** | **Factor 3** | **Factor 4** |
| **Emotional Lability** | **0.73** | 0.00 | -0.06 | 0.19 |
| **Anxiousness** | **0.75** | -0.05 | 0.17 | **0.30** |
| **Separation Insecurity** | **0.62** | 0.11 | 0.00 | 0.21 |
| **Submissiveness** | **0.48** | 0.16 | 0.12 | 0.04 |
| **Hostility** | **0.43** | **0.37** | 0.29 | 0.23 |
| **Perseveration** | **0.62** | 0.20 | **0.32** | **0.33** |
| **Withdrawal** | 0.26 | 0.10 | **0.78** | 0.19 |
| **Intimacy Avoidance** | 0.01 | 0.07 | **0.55** | 0.04 |
| **Anhedonia** | **0.56** | 0.00 | **0.64** | -0.03 |
| **Depressivity** | **0.67** | 0.11 | **0.51** | -0.04 |
| **Restricted Affectivity** | 0.06 | 0.29 | **0.65** | 0.16 |
| **Suspiciousness** | **0.43** | 0.21 | 0.27 | 0.25 |
| **Manipulativeness** | 0.20 | **0.78** | 0.08 | 0.17 |
| **Deceitfulness** | 0.25 | **0.75** | 0.22 | 0.02 |
| **Grandiosity** | 0.08 | **0.52** | 0.13 | **0.47** |
| **Attention Seeking** | **0.41** | **0.56** | -0.07 | 0.22 |
| **Callousness** | 0.12 | **0.60** | **0.51** | 0.08 |
| **Irresponsibility** | **0.41** | **0.58** | 0.28 | -0.19 |
| **Impulsivity** | **0.52** | **0.30** | 0.04 | -0.07 |
| **Distractibility** | **0.65** | 0.24 | 0.25 | -0.07 |
| **Risk Taking** | -0.04 | **0.49** | 0.06 | -0.06 |
| **Rigid Perfectionism** | 0.23 | -0.05 | 0.16 | **0.69** |
| **Unusual Beliefs & Experiences** | **0.33** | **0.33** | 0.19 | **0.35** |
| **Eccentricity** | **0.41** | **0.37** | **0.46** | 0.19 |
| **Perceptual Dysregulation** | **0.61** | **0.34** | **0.31** | 0.25 |

|  | **6-factor Model** | | | | | |
| --- | --- | --- | --- | --- | --- | --- |
| **Facets** | **Factor 1** | **Factor 2** | **Factor 3** | **Factor 4** | **Factor 5** | **Factor 6** |
| **Emotional Lability** | **0.59** | -0.27 | -0.28 | 0.11 | 0.21 | 0.35 |
| **Anxiousness** | **0.75** | -0.09 | 0.00 | 0.23 | -0.07 | 0.11 |
| **Separation Insecurity** | **0.60** | 0.11 | -0.18 | 0.14 | -0.08 | 0.14 |
| **Submissiveness** | **0.63** | 0.28 | -0.04 | -0.04 | -0.11 | -0.18 |
| **Hostility** | 0.07 | 0.13 | 0.22 | 0.15 | -0.19 | **0.70** |
| **Perseveration** | **0.51** | 0.07 | 0.15 | 0.23 | 0.16 | 0.00 |
| **Withdrawal** | 0.15 | -0.07 | **0.80** | 0.14 | -0.06 | 0.03 |
| **Intimacy Avoidance** | -0.09 | -0.11 | **0.60** | 0.02 | 0.08 | -0.02 |
| **Anhedonia** | **0.59** | -0.14 | **0.58** | -0.10 | -0.12 | 0.00 |
| **Depressivity** | **0.69** | -0.07 | 0.36 | -0.15 | 0.06 | -0.04 |
| **Restricted Affectivity** | -0.09 | 0.15 | **0.67** | 0.10 | 0.06 | -0.03 |
| **Suspiciousness** | 0.29 | 0.09 | 0.17 | 0.18 | -0.04 | 0.21 |
| **Manipulativeness** | 0.06 | **0.89** | -0.10 | 0.05 | 0.03 | -0.06 |
| **Deceitfulness** | 0.15 | **0.82** | 0.06 | -0.13 | -0.06 | -0.03 |
| **Grandiosity** | -0.14 | **0.53** | 0.04 | 0.40 | 0.14 | 0.01 |
| **Attention Seeking** | 0.31 | **0.61** | -0.28 | 0.10 | 0.06 | 0.01 |
| **Callousness** | -0.18 | 0.41 | **0.49** | -0.03 | -0.05 | 0.29 |
| **Irresponsibility** | 0.27 | 0.37 | 0.11 | -0.33 | 0.13 | 0.11 |
| **Impulsivity** | 0.29 | -0.04 | -0.14 | -0.22 | 0.07 | **0.59** |
| **Distractibility** | **0.60** | 0.06 | 0.06 | -0.19 | 0.09 | 0.10 |
| **Risk Taking** | -0.30 | 0.25 | -0.02 | -0.16 | 0.39 | 0.12 |
| **Rigid Perfectionism** | 0.13 | -0.04 | 0.11 | **0.66** | 0.13 | -0.06 |
| **Unusual Beliefs & Experiences** | 0.06 | 0.02 | 0.01 | 0.29 | **0.70** | -0.09 |
| **Eccentricity** | 0.18 | 0.02 | 0.31 | 0.07 | **0.54** | -0.06 |
| **Perceptual Dysregulation** | 0.43 | 0.02 | 0.07 | 0.13 | **0.57** | -0.08 |

Supplementary Material SM8: PID-5 (paper-and-pencil) Hierarchical Structure (N=730)

| **Level 1** | | |  |
| --- | --- | --- | --- |
| **Facets** | **Factor 1** |  |  |
| **Emotional Lability** | 0.51 |  |  |
| **Anxiousness** | 0.64 |  |  |
| **Separation Insecurity** | 0.53 |  |  |
| **Submissiveness** | 0.48 |  |  |
| **Hostility** | 0.68 |  |  |
| **Perseveration** | 0.76 |  |  |
| **Withdrawal** | 0.63 |  |  |
| **Intimacy Avoidance** | 0.30 |  |  |
| **Anhedonia** | 0.66 |  |  |
| **Depressivity** | 0.74 |  |  |
| **Restricted Affectivity** | 0.52 |  |  |
| **Suspiciousness** | 0.60 |  |  |
| **Manipulativeness** | 0.58 |  |  |
| **Deceitfulness** | 0.63 |  |  |
| **Grandiosity** | 0.48 |  |  |
| **Attention Seeking** | 0.53 |  |  |
| **Callousness** | 0.62 |  |  |
| **Irresponsibility** | 0.64 |  |  |
| **Impulsivity** | 0.51 |  |  |
| **Distractibility** | 0.66 |  |  |
| **Risk Taking** | <0.30 |  |  |
| **Rigid Perfectionism** | 0.37 |  |  |
| **Unusual Beliefs & Experiences** | 0.57 |  |  |
| **Eccentricity** | 0.74 |  |  |
| **Perceptual Dysregulation** | 0.80 |  |  |
| **Level 2** | | | |
| **Facets** | **Factor 1** | **Factor 2** | |
| **Emotional Lability** | **0.81** | -0.28 | |
| **Anxiousness** | **0.97** | **-0.30** | |
| **Separation Insecurity** | **0.67** | -0.11 | |
| **Submissiveness** | **0.48** | 0.03 | |
| **Hostility** | **0.39** | **0.36** | |
| **Perseveration** | **0.73** | 0.09 | |
| **Withdrawal** | **0.43** | 0.26 | |
| **Intimacy Avoidance** | 0.10 | 0.24 | |
| **Anhedonia** | **0.69** | 0.02 | |
| **Depressivity** | **0.75** | 0.05 | |
| **Restricted Affectivity** | 0.11 | **0.50** | |
| **Suspiciousness** | **0.49** | 0.16 | |
| **Manipulativeness** | -0.12 | **0.83** | |
| **Deceitfulness** | -0.08 | **0.85** | |
| **Grandiosity** | 0.00 | **0.57** | |
| **Attention Seeking** | 0.20 | **0.44** | |
| **Callousness** | -0.06 | **0.81** | |
| **Irresponsibility** | 0.15 | **0.59** | |
| **Impulsivity** | **0.39** | **0.17** | |
| **Distractibility** | **0.60** | 0.12 | |
| **Risk Taking** | **-0.32** | **0.61** | |
| **Rigid Perfectionism** | **0.47** | -0.08 | |
| **Unusual Beliefs & Experiences** | **0.32** | **0.32** | |
| **Eccentricity** | 0.40 | **0.42** | |
| **Perceptual Dysregulation** | **0.63** | 0.26 | |

| **Level 3** | | | |
| --- | --- | --- | --- |
| **Facets** | **Factor 1** | **Factor 2** | **Factor 3** |
| **Emotional Lability** | **0.93** | -0.27 | -0.15 |
| **Anxiousness** | **0.94** | -0.01 | -0.26 |
| **Separation Insecurity** | **0.75** | -0.20 | 0.00 |
| **Submissiveness** | **0.49** | -0.02 | 0.06 |
| **Hostility** | **0.36** | 0.15 | 0.29 |
| **Perseveration** | **0.66** | 0.17 | 0.04 |
| **Withdrawal** | 0.12 | **0.83** | -0.11 |
| **Intimacy Avoidance** | -0.16 | **0.64** | -0.04 |
| **Anhedonia** | **0.46** | **0.60** | -0.25 |
| **Depressivity** | **0.59** | 0.40 | -0.12 |
| **Restricted Affectivity** | -0.16 | **0.70** | 0.20 |
| **Suspiciousness** | **0.44** | 0.16 | 0.10 |
| **Manipulativeness** | 0.00 | -0.13 | **0.89** |
| **Deceitfulness** | -0.01 | 0.05 | **0.80** |
| **Grandiosity** | 0.05 | 0.01 | **0.55** |
| **Attention Seeking** | **0.38** | **-0.31** | **0.60** |
| **Callousness** | -0.18 | **0.45** | **0.58** |
| **Irresponsibility** | 0.16 | 0.13 | **0.51** |
| **Impulsivity** | **0.47** | -0.13 | 0.22 |
| **Distractibility** | **0.58** | 0.09 | 0.09 |
| **Risk Taking** | -0.25 | -0.01 | **0.58** |
| **Rigid Perfectionism** | **0.41** | 0.09 | -0.10 |
| **Unusual Beliefs & Experiences** | **0.33** | 0.06 | 0.29 |
| **Eccentricity** | 0.29 | **0.35** | 0.26 |
| **Perceptual Dysregulation** | **0.60** | 0.13 | 0.20 |

|  |  | **Level 4** |  |  |
| --- | --- | --- | --- | --- |
| **Facets** | **Factor 1** | **Factor 2** | **Factor 3** | **Factor 4** |
| **Emotional Lability** | **0.87** | -0.26 | -0.17 | 0.11 |
| **Anxiousness** | **0.83** | 0.01 | -0.29 | 0.22 |
| **Separation Insecurity** | **0.69** | -0.19 | -0.03 | 0.14 |
| **Submissiveness** | **0.52** | -0.01 | 0.05 | -0.04 |
| **Hostility** | **0.31** | 0.15 | 0.26 | 0.15 |
| **Perseveration** | **0.56** | 0.18 | 0.00 | 0.25 |
| **Withdrawal** | 0.06 | **0.83** | -0.12 | 0.14 |
| **Intimacy Avoidance** | -0.16 | **0.63** | -0.04 | 0.02 |
| **Anhedonia** | **0.53** | **0.62** | -0.25 | -0.13 |
| **Depressivity** | **0.68** | **0.41** | -0.12 | -0.17 |
| **Restricted Affectivity** | -0.20 | **0.69** | 0.18 | 0.12 |
| **Suspiciousness** | **0.36** | 0.16 | 0.06 | 0.18 |
| **Manipulativeness** | 0.01 | -0.12 | **0.84** | 0.08 |
| **Deceitfulness** | 0.08 | 0.05 | **0.79** | -0.10 |
| **Grandiosity** | -0.15 | 0.01 | **0.53** | **0.45** |
| **Attention Seeking** | **0.35** | **-0.30** | **0.56** | 0.13 |
| **Callousness** | -0.14 | **0.45** | **0.57** | 0.00 |
| **Irresponsibility** | **0.34** | 0.13 | **0.55** | **-0.33** |
| **Impulsivity** | **0.58** | -0.13 | 0.23 | -0.18 |
| **Distractibility** | **0.70** | 0.09 | 0.09 | -0.20 |
| **Risk Taking** | -0.17 | -0.01 | **0.58** | -0.11 |
| **Rigid Perfectionism** | 0.11 | 0.11 | -0.22 | **0.72** |
| **Unusual Beliefs & Experiences** | 0.21 | 0.07 | 0.24 | **0.30** |
| **Eccentricity** | 0.27 | **0.36** | 0.23 | 0.09 |
| **Perceptual Dysregulation** | **0.55** | 0.13 | 0.17 | 0.14 |

| **Level 5** | | | | | |
| --- | --- | --- | --- | --- | --- |
| **Facets** | **Factor 1** | **Factor 2** | **Factor 3** | **Factor 4** | **Factor 5** |
| **Emotional Lability** | **0.83** | -0.29 | 0.23 | 0.11 | -0.24 |
| **Anxiousness** | **0.83** | 0.01 | -0.14 | 0.22 | -0.10 |
| **Separation Insecurity** | **0.69** | -0.18 | -0.11 | 0.13 | 0.12 |
| **Submissiveness** | **0.53** | 0.00 | -0.20 | -0.04 | 0.20 |
| **Hostility** | **0.31** | 0.16 | 0.05 | 0.13 | 0.28 |
| **Perseveration** | **0.54** | 0.17 | 0.07 | 0.23 | 0.04 |
| **Withdrawal** | 0.09 | **0.82** | -0.08 | 0.14 | -0.04 |
| **Intimacy Avoidance** | -0.15 | **0.62** | 0.07 | 0.02 | -0.09 |
| **Anhedonia** | **0.55** | **0.62** | -0.17 | -0.11 | -0.15 |
| **Depressivity** | **0.67** | **0.40** | -0.01 | -0.15 | -0.12 |
| **Restricted Affectivity** | -0.18 | **0.69** | 0.05 | 0.10 | 0.17 |
| **Suspiciousness** | **0.36** | 0.17 | -0.02 | 0.17 | 0.14 |
| **Manipulativeness** | 0.00 | -0.10 | 0.03 | 0.05 | **0.87** |
| **Deceitfulness** | 0.07 | 0.07 | -0.04 | -0.13 | **0.82** |
| **Grandiosity** | -0.15 | 0.02 | 0.13 | **0.40** | **0.56** |
| **Attention Seeking** | **0.33** | -0.28 | 0.04 | 0.10 | **0.59** |
| **Callousness** | -0.13 | **0.46** | 0.09 | -0.03 | **0.50** |
| **Irresponsibility** | **0.30** | 0.13 | 0.18 | **-0.33** | **0.37** |
| **Impulsivity** | **0.53** | -0.15 | 0.22 | -0.18 | 0.08 |
| **Distractibility** | **0.66** | 0.08 | 0.07 | -0.19 | 0.04 |
| **Risk Taking** | -0.23 | -0.03 | **0.47** | -0.15 | 0.27 |
| **Rigid Perfectionism** | 0.13 | 0.11 | 0.02 | **0.67** | -0.04 |
| **Unusual Beliefs & Experiences** | 0.12 | 0.03 | **0.59** | **0.30** | -0.02 |
| **Eccentricity** | 0.21 | **0.34** | **0.47** | 0.08 | -0.02 |
| **Perceptual Dysregulation** | **0.49** | 0.10 | **0.45** | 0.13 | -0.04 |
